# Study on viscosity time-varying law and microscopic chain forming mechanism of Fe_3_O_4_ magnetic slurry

**DOI:** 10.1371/journal.pone.0354348

**Published:** 2026-08-24

**Authors:** Zheng Li, Changbai Shi, Anbang Pan, Yuming He, Mingxin Liu, Lei Liu, Rucheng Wang

**Affiliations:** 1 Hubei Key Laboratory of Resource and Eco-environmental Geology, Hubei Geological Bureau, Wuhan, China; 2 Hydrogeology and Engineering Geology Institute of Hubei Geological Bureau, Jingzhou, China; University of Central Florida, UNITED STATES OF AMERICA

## Abstract

Complex and changeable engineering geological conditions pose major challenges to underwater construction. Slurry grouting under dynamic water conditions is difficult to regulate in real time, resulting in slurry loss and a low retention rate. This study develops a new type of magnetic grouting material based on materials science and electromagnetism, and examines the characteristics of slurry with different magnetic powder dosages. The relationship between shear stress and magnetic field strength was investigated using a rheological test. The magnetisation rate (*χ*) of the material was measured using a vibrating sample magnetometer (VSM), and the distribution pattern of the magnetic chains in the slurry was observed using a CT scan and an optical microscope. The retention rate of the slurry in moving water was tested using an anti-scour test. The results show that the magnetisation rate of the slurry increases linearly with an increase in magnetic powder doping, the magnetisation rate with 35% magnetic powder doping is 6. 89 times that with 5%. The rheological curve of the slurry conforms to the Bingham model and the dynamic yield stress increases with an increase in magnetic field strength. Magnetic particles are arranged in short chain, long chain, double chain, and bundle chain forms. When the water flow velocity is 0. 8m/s, the retention rate of the slurry is increased by 8. 68 times compared to ordinary slurry. This study provides theoretical guidance for the mechanism of magnetic slurry.

## 1. Introduction

In the domain of grouting engineering, the issue of high-pressure water surge is becoming increasingly significant. This phenomenon poses a threat not only to the safety of construction personnel but also results in significant economic losses [1–4]. The complexity of the engineering geological structure gives rise to a substantial number of gushing water fractures, which are inclined at a significant angle. This phenomenon is the cause of a significant loss of slurry along the fractures during the grouting process [5,6]. During the grouting process, rheological parameters such as apparent viscosity, yield stress, plasticity and thixotropy of the slurry are of greater significance [7,8]. The rheological properties of the slurry are pivotal in determining its pumpability, with a slurry exhibiting optimal fluidity possessing a broad diffusion area within the fissure and a high filling rate [9].

The application of magnetorheological materials in the field of concrete is primarily concerned with the enhancement of concrete properties, including pumpability, de-icing effect, frost resistance, durability, and elongation properties. In early research, the hydration activity of cement particles was modified by incorporating magnetised water into concrete. This marked the early application of magnetic materials in the concrete industry [10–12]. Subsequently, Zhao et al. [13] investigated the preparation conditions of magnetised water under an alternating magnetic field with the aim of obtaining the optimum reinforcement of cement mortar. Choi et al. [14] activated the lubricating layer of water in concrete pumping pipelines by electromagnetic field and changed its properties to improve the pumpability. Wang et al. [15] incorporated magnetite as a coarse aggregate into concrete, which significantly enhanced the microwave deicing effect, freezing resistance and durability of the concrete. The elongation of concrete have been enhanced by researchers through the application of a magnetic field, which has enabled the orientation of steel fibres within the concrete [16]. The findings of the study demonstrated that, within a vertical magnetic field, steel fibres exhibited an aggregation tendency in the central region. Conversely, within a horizontal magnetic field, steel fibres demonstrated an aggregation tendency towards the sides [17].

In the field of mechanical property enhancement of cementitious materials, researchers have incorporated magnetic particles into the cement matrix with the objective of enhancing its mechanical and other properties. Liu et al. [18] developed a magnetic grouting material whose viscosity can be modulated transiently, maintained within a fixed area, and is capable of automatically discharging gases and water. Li et al. [19] examined the mechanical properties of nano-Fe_2_O_3_ cement mortar and the strain self-sensing properties, ascertaining that it possesses the capacity to sense its own stress and can be utilised as a smart structural material. Gurram et al. [20] incorporated a high-performance magnetic powder (NdFeB) into cement-based blends, thereby achieving substantial enhancement in the mechanical properties of the resultant cement pastes. This integration also resulted in a significant enhancement of the density and compressive strength of the magnetic cement composites. In a related study, Dong et al. [21] investigated the mechanism through which Fe_3_O_4_ affects cementitious materials, determining that it can remarkably improve the mechanical properties and durable impermeability of cement pastes. It has been established by other researchers that the application of a static magnetic field can facilitate molecular reorganisation during the hydration of cement, thereby enhancing the microstructure of cement paste and reducing porosity. In addition, the incorporation of 3% magnetite nanoparticles into cement mortar has been demonstrated to result in a substantial augmentation in compressive strength [22,23].

In the domain of electrical properties of cementitious materials, the electrical and mechanical properties of cementitious materials are predominantly enhanced by the incorporation of nickel powders and their subsequent orientation. Xiao et al. [24] incorporated nickel powders into the cementitious material, observing a decline in resistivity with increasing nickel powder content. The orientation of nickel powders within the slurry, resulting from the addition of nickel powders, was found to impart exceptional signal transmission properties to the material. Tian et al. [25]conducted a study into the electrical and mechanical properties of magnetically chain-forming nickel powder-oriented aligned cementitious composites. The issue of the random dispersion of nickel powder particles was resolved.

In the study of the effect of magnetic field on rheological properties, Hu et al. [26,27] investigated the effect of horizontal and vertical magnetic fields on the rheological properties of ferric oxide magnetic fluid with different morphology. The study found that the magnetic fluid exhibits different fluid properties (Newtonian or Bingham fluid) in response to varying magnetic field directions and intensities, and that magnetic particle morphology has an effect on the magnetic moment transfer. Liu et al. [28] investigated the effects of temperature, magnetic field strength and magnetic particle doping on the viscosity of magnetic fluids. It was found that 30% magnetic particle doping was the critical point for the rate of change of viscosity, and the temperature had a greater influence on the change of viscosity in the presence of a magnetic field. Anupam et al. [29] investigated the effect of the diameter of the magnetic particles and the thickness of the surfactant layer on the viscosity of ferromagnetic fluids. The study found that increasing the diameter of the particles led to a reduction in viscosity, while increasing the thickness of the surfactant layer resulted in enhanced the viscosity. The viscosity is enhanced by a static magnetic field, and the viscosity under an alternating magnetic field is related to both the magnetic field strength and frequency. Qiu et al. [30] established a magnetorheological mechanical model of ferromagnetic particles with different particle sizes from a theoretical point of view, and tested the shear properties of magnetorheological. Zhang et al. [31] investigated the effect of cement-to-sand (c/s) ratio and sand gradation on the magnetorheological response of cement mortar. Some researchers have investigated the effects of the magnetorheological effect on the early hydration products, microstructure, and early compressive strength of cement paste containing Fe_3_O_4_ [32–34].

Although previous studies have examined the role of magnetic materials in modulating the rheological properties of cement paste, the mechanism underlying the time-dependent viscosity of cement paste under a magnetic field remains unclear. At the microscopic level, there remains a lack of systematic theoretical explanations for the formation mechanisms of magnetic particle chains and their contribution to changes in slurry viscosity. Relevant work has not yet clarified the distribution patterns of magnetic particles within the slurry or the formation mechanism of the slurry’s shear strength. This study aims to systematically investigate the influence of Fe_3_O_4_ on the rheological properties of cement slurry, reveal the time-dependent characteristics of slurry viscosity under the influence of a magnetic field, and elucidate the mechanism of viscosity changes from the perspective of the microstructure of magnetic particle chains, with the goal of providing a theoretical basis for the engineering applications of cement-based magnetic slurries.

## 2. Materials and methods

### 2.1. Materials

#### 2.1.1. Fe_3_O_4_.

**Fig 1:**
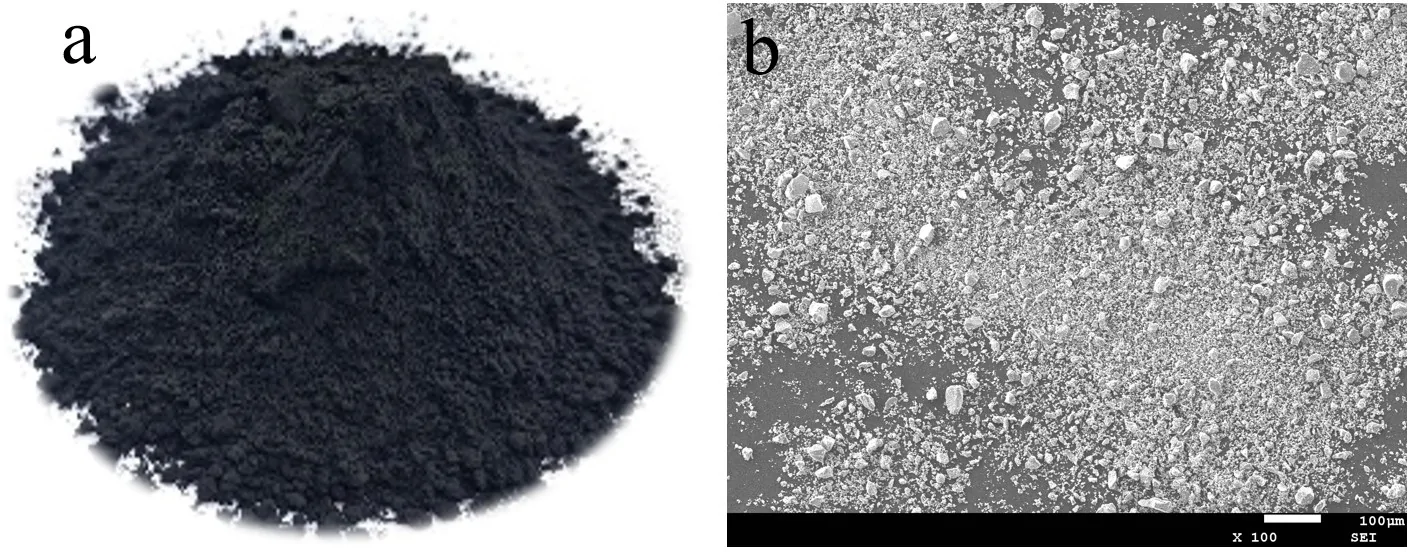
(a) Fe_3_O_4_ physical picture; (b) Micro SEM image of Fe_3_O_4_. Fe_3_O_4_.

**Fig 2:**
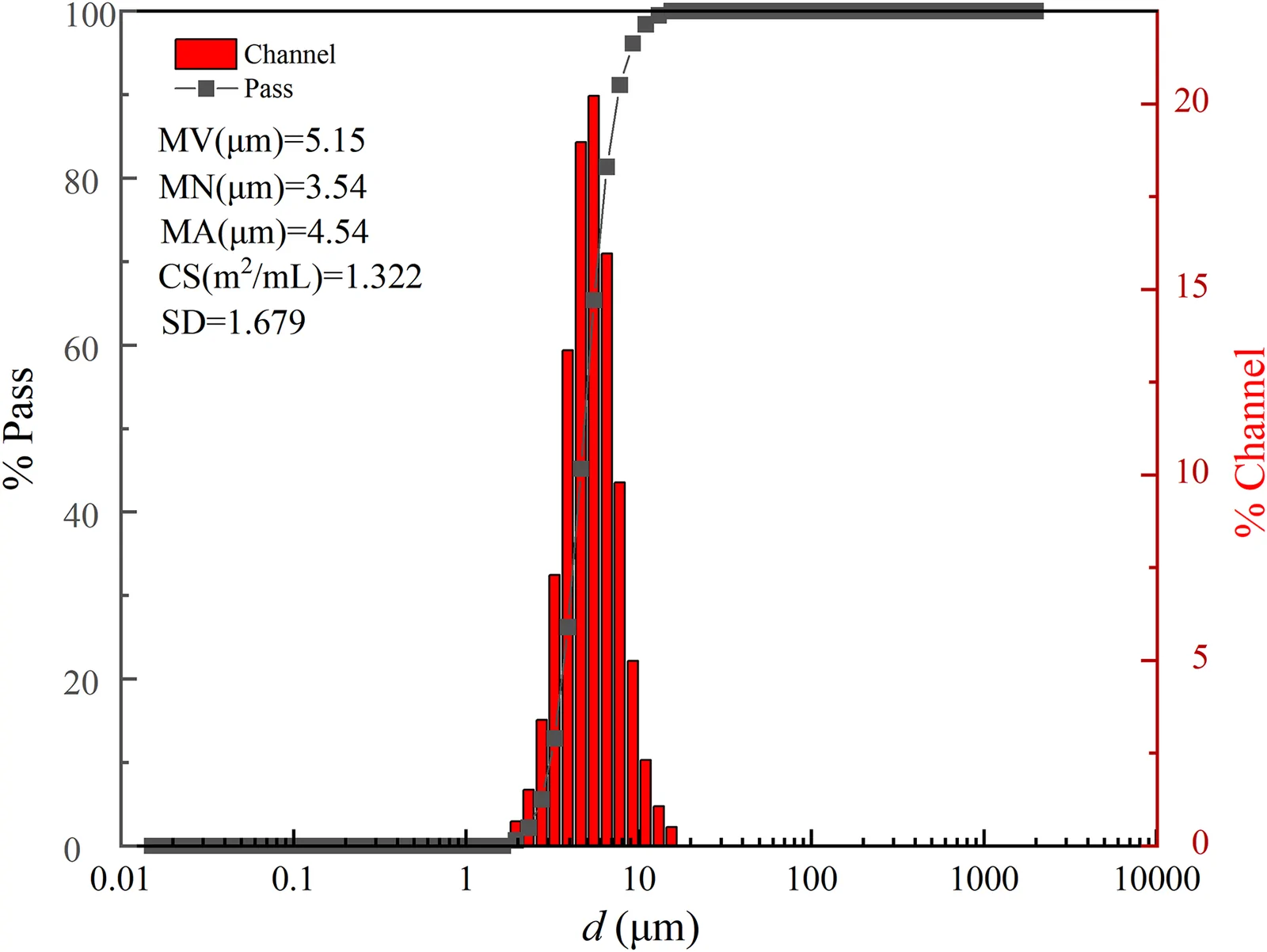
Particle size distribution curve of Fe_3_O_4_.

**Fig 3:**
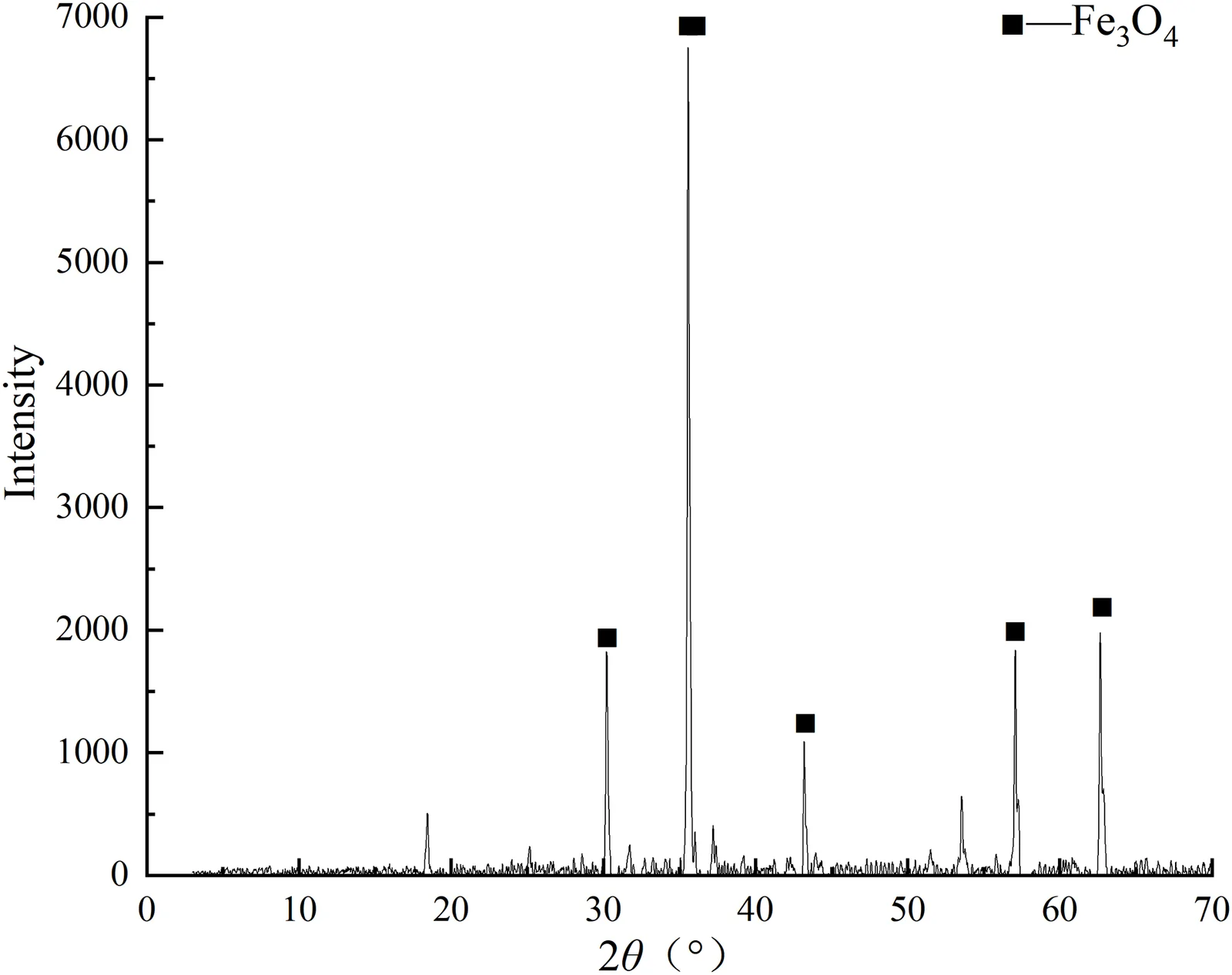
XRD pattern of Fe_3_O_4_.

As shown in Fig 1, the Fe_3_O_4_ has the appearance of a black powder and the microscopic morphology of an irregular ribbed mass. It has properties such as high specific gravity and strong magnetic properties. The Fe_3_O_4_ particles have superparamagnetic properties, showing magnetic properties when an applied magnetic field is added and disappearing when the applied field is removed. The Fig 2 shows the size distribution curve of the magnetic particles with a mean particle size (MN) of 3.54 μm, where D10 = 8.8 μm, D50 = 4.82 μm and D90 = 7.58 μm. In Fig 3, the magnetic particle XRD diffraction pattern demonstrated its most intense peaks at 35°.

#### 2.1.2. Cement.

**Fig 4:**
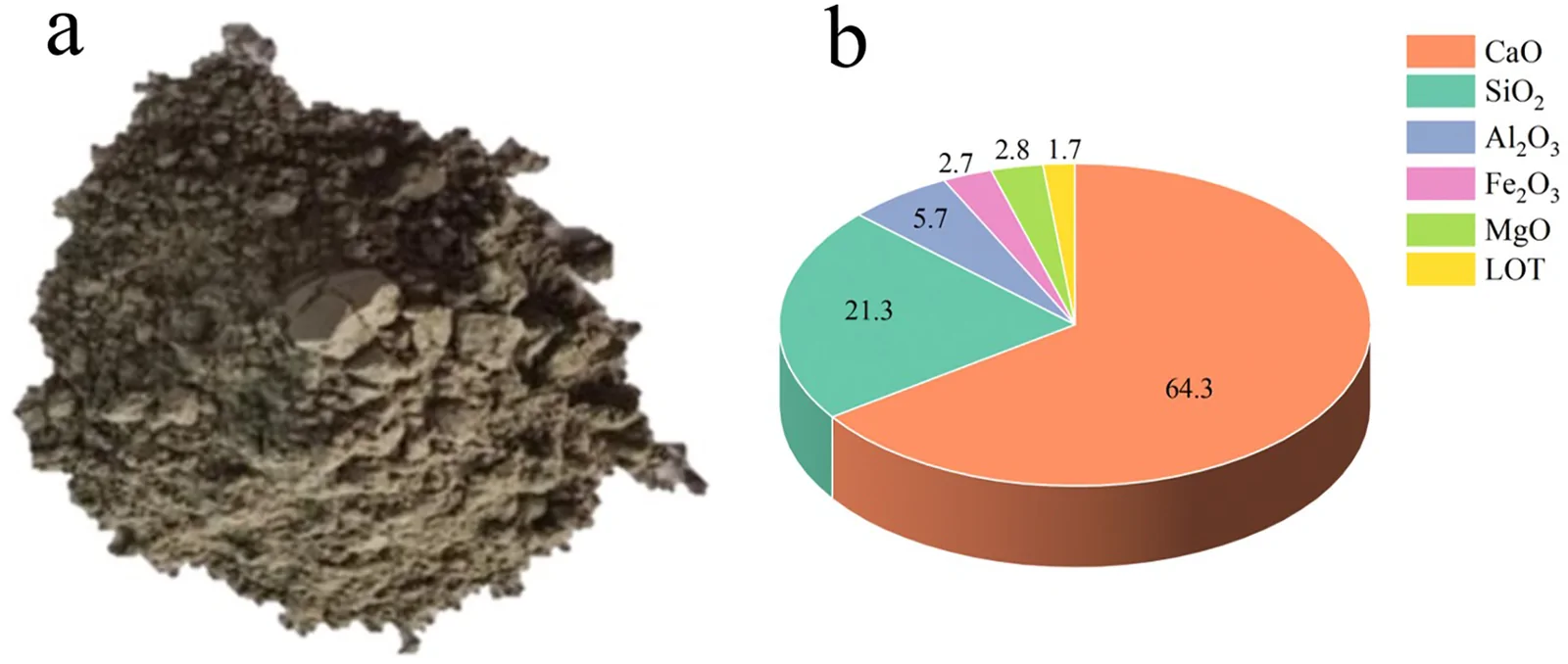
(a) Cement photograph. (b) Chemical composition (%) of the OPC. Ordinary portland cement.

**Fig 5:**
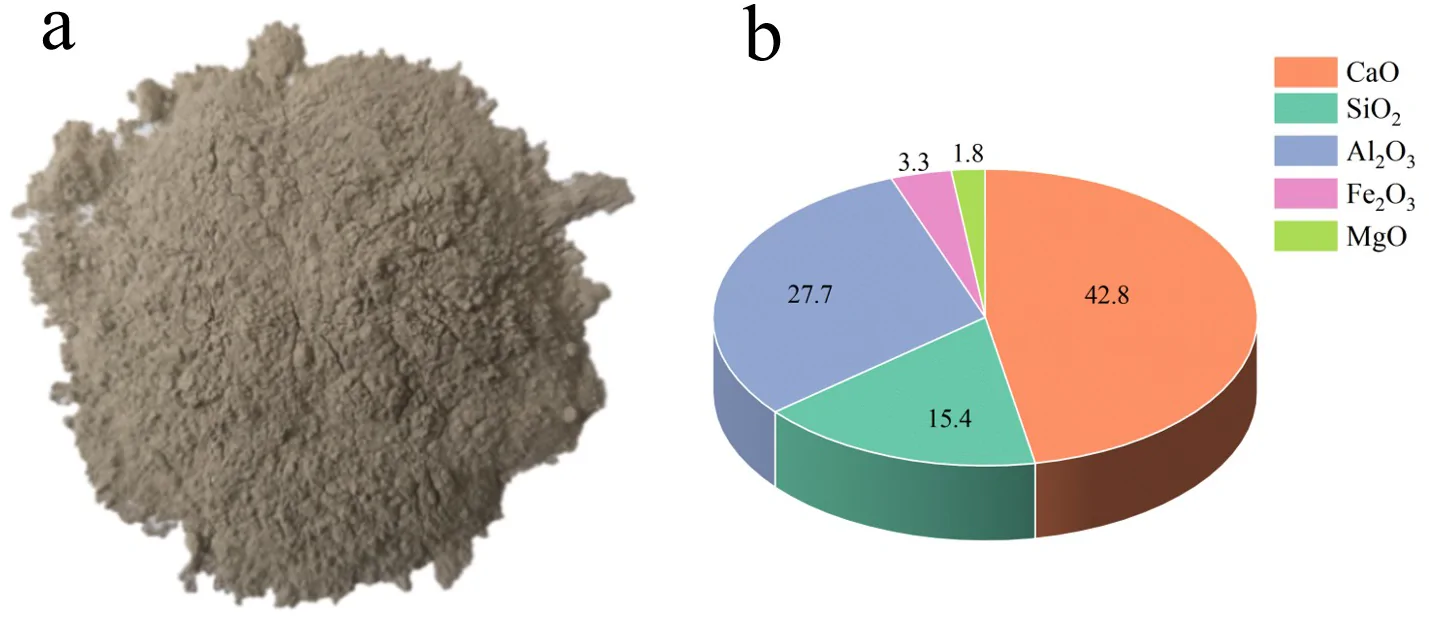
(a) Cement photograph. (b) Chemical composition (%) of the SAC. Aluminosilicate cement.

The cement used is a composite cement consisting of ordinary Portland cement with a strength grade of 42.5 and sulfoaluminate cement, with the sulfoaluminate cement accounting for 30% of the total cement content. As shown in Figs 4 and 5, ordinary portland cement is greyish-green in colour, whilst sulphaluminate cement is light grey. The aluminosilicate cement, also known as fast-setting cement, is characterised by its rapid setting rate and the ability to adjust its initial and final setting times. The predominant components were found to be calcium oxide (CaO) and silicon dioxide (SiO_2_).

#### 2.1.3. Water-based epoxy resin emulsion (Model: DY-128–50).

**Fig 6:**
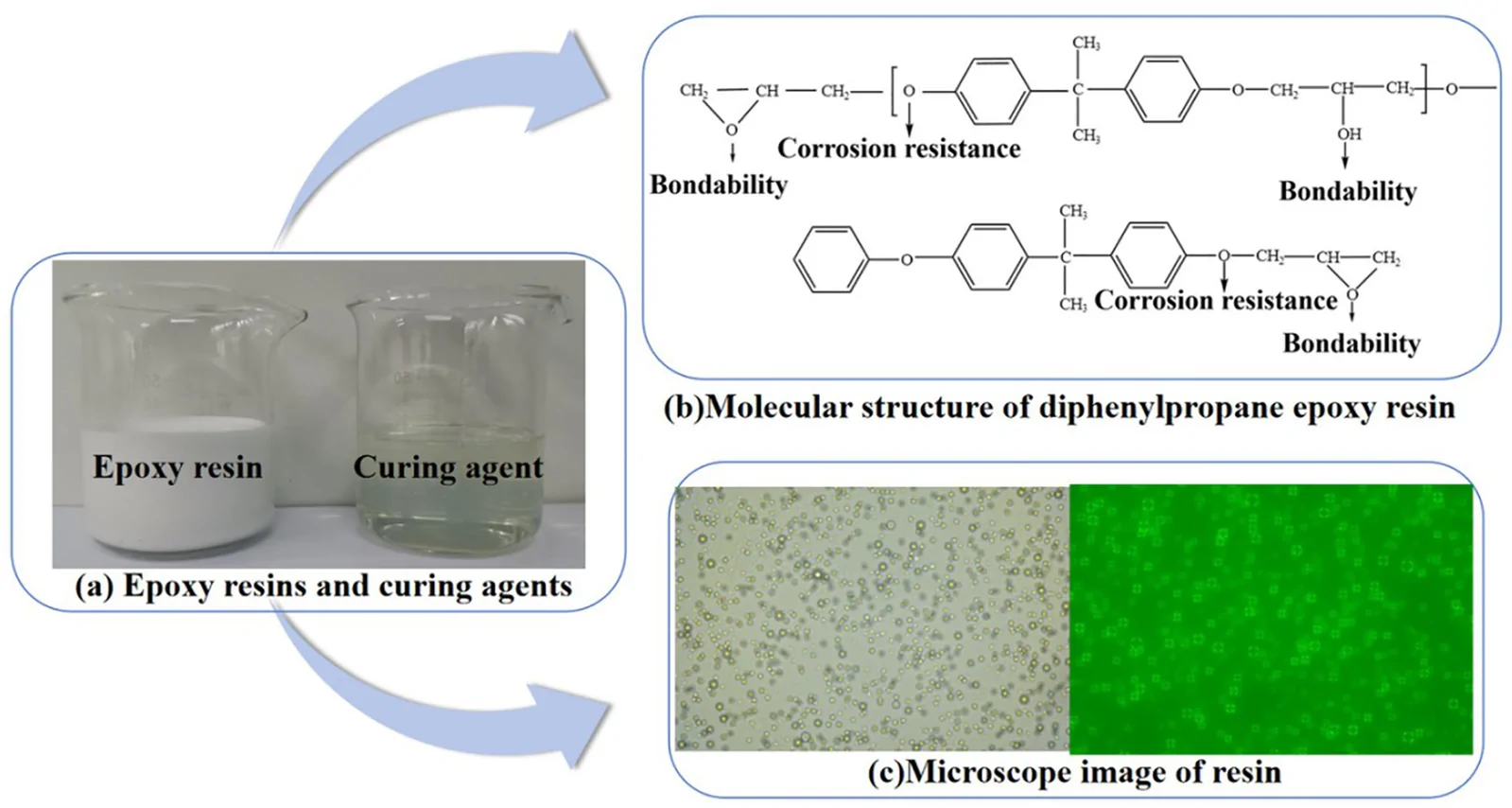
Microscope image of resin.

The water-based epoxy resin is E-51 type epoxy resin, the main constituent substances are diphenol propane and epichlorohydrin, and the epoxy value index is 0. 52 mol/100g. It boasts excellent water and abrasion resistance, as well as high flexibility and impact resistance. As shown in Fig 6, the epoxy resin particles were small, spherical and uniformly dispersed in the solution. There was no aggregation or stacking between the particles.

### 2.2. Experimental apparatus

#### 2.2.1. Magnetisation rate test.

The vibrating sample magnetometer (VSM) is used to measure a variety of magnetic performance parameters of ferromagnetic, paramagnetic and other materials, such as coercivity Hc, saturation magnetization Ms, residual magnetization Mr, etc.

#### 2.2.2. Rheological testing.

This study used an LVDV-2T infinitely adjustable rotary viscometer for the measurements. The electromagnets are placed parallel to each other so that the direction of the magnetic field strength is perpendicular to the rotor. The magnetic slurry was in direct contact with the rotating rotor. The initial and test temperatures were 26℃. Testing commenced immediately after stirring had finished, with a shear rate of 0–41. 8 s^-1^. To investigate the variation in shear stress and viscosity of the slurry at different magnetic powder loadings (5%, 10%, 15% and 20%). Additionally, the viscosity transient effect of the slurry was tested under magnetic field intensities ranging from 0 GS to 500GS. The viscosity transient effect was tested separately for each magnetic field strength (0GS, 100GS, 200GS, 300 GS, 400GS, and 500GS).

#### 2.2.3. Magneto-chaining test.

To facilitate observation and research, a transparent resin with a viscosity matching that of the cement slurry was selected as a substitute for the cement slurry. On this basis, magnetic particles were uniformly mixed into the transparent resin. The experiment involved placing the slurry on a slide and recording the magnetic chains moved and changed shape. This was achieved by attaching a magnet to the microscope stage and employing a combination of reflective and transmissive photography techniques with a high-speed, high-definition digital camera that was connected to the microscope.

#### 2.2.4. Slurry scour resistance test.

The dynamic grouting process is simulated using the direct pouring test method, whereby the mixed grout is poured at a constant rate 20 cm from the inlet. The total mass of the grout and the beaker before pouring is denoted as *M*_0_, and the mass of the beaker after pouring is denoted as m. When the grout at the outlet is no longer turbid, the pressure pump and the inlet valve are shut off, and the mass of the remaining grout in the tank is weighed and denoted as *M*. The formula for calculating the grout retention rate *η* is as follows:


$$\begin{array}{c} \eta =\frac{M}{M_{\text{0}}-m} \end{array}$$ (1)

In the formula: *M* represents the remaining slurry mass (g);

*M*_0_ is the total mass (g) of the slurry and beaker before pouring;

*m* is the mass of the beaker after pouring (g).

## 3. Results and discussion

### 3.1. Magnetisation characterisation of slurry

**Fig 7:**
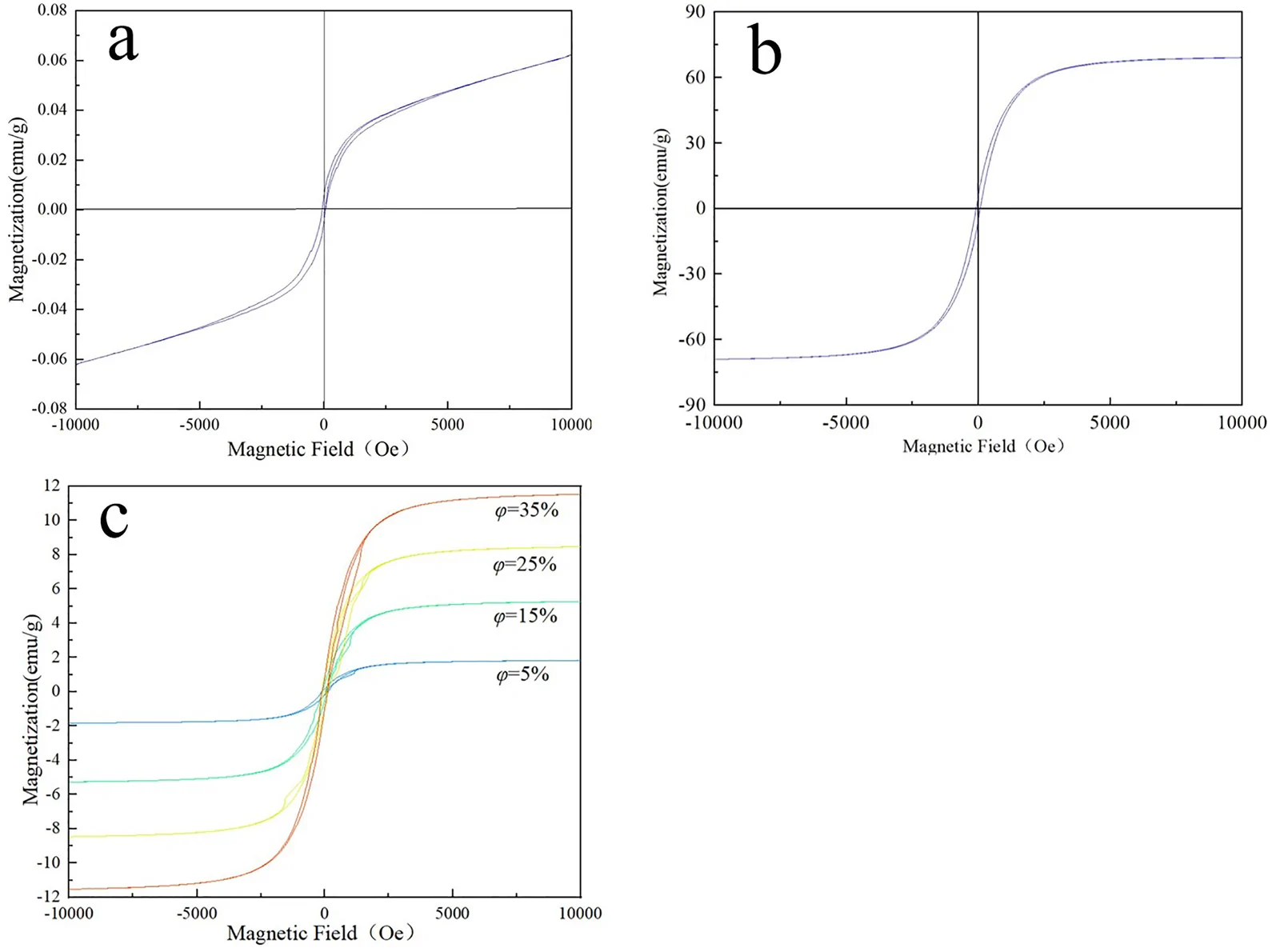
(a) Cement. **(b) Fe**_**3**_**O**_**4**_**. (c) MEC slurry.** Magnetisation curve.

As shown in Fig 7, the magnetisation strength of the magnetic slurry reaches saturation when the applied magnetic field increases from zero. The saturation magnetisation strength increases with the addition of magnetic powder. The hysteresis return line of the magnetic slurry is relatively narrow. This means that it is easily magnetised and demagnetised, and that the hysteresis loss is relatively small. It therefore belongs to the soft magnetic class of materials. The slurry with 35% magnetic powder doping has the highest saturation magnetisation strength and mass magnetisation rate.

**Fig 8:**
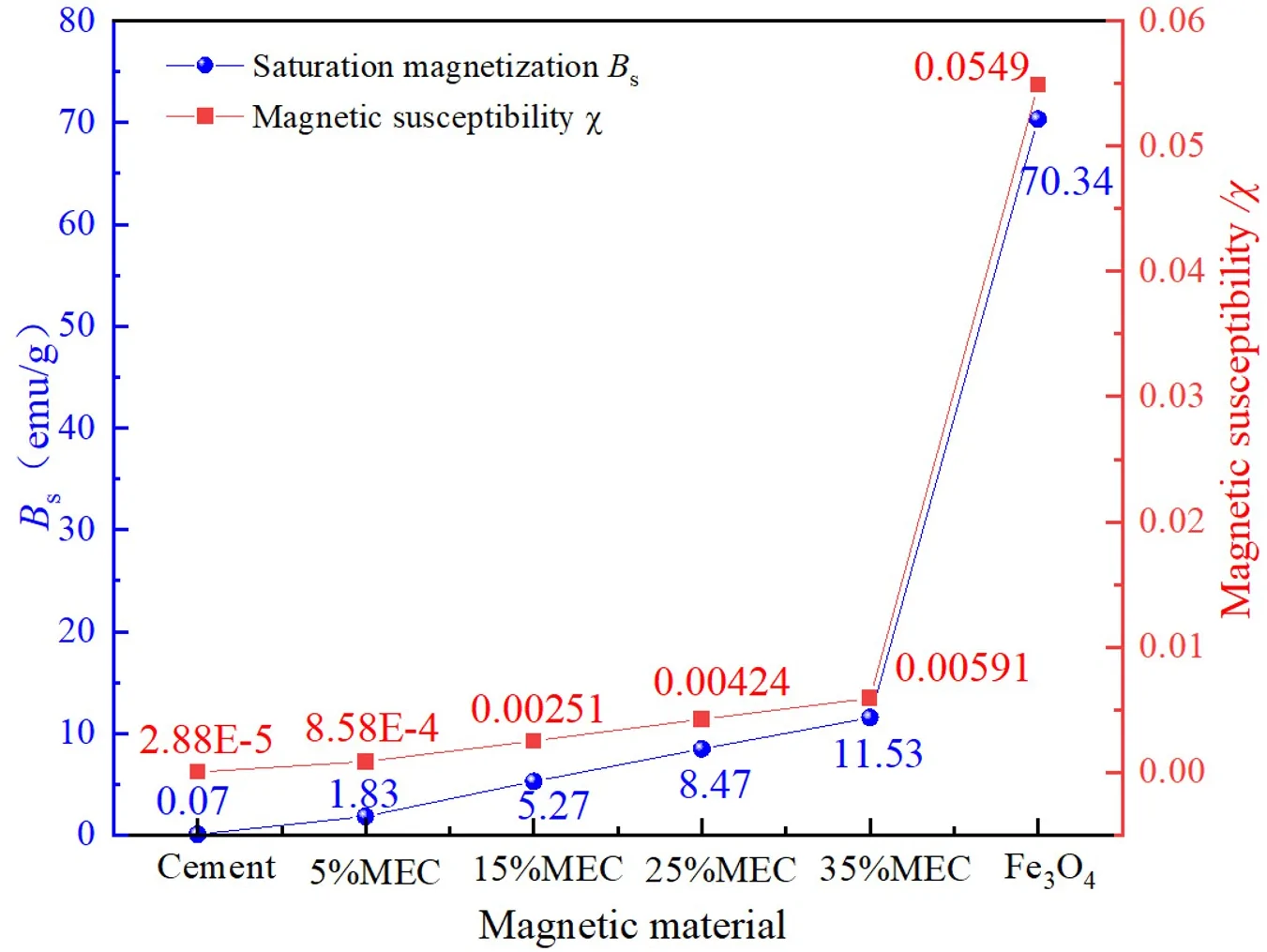
Saturation magnetisation strength and magnetisation rate.

As shown in Table 1, the magnetic parameters of Fe_3_O_4_, composite cement and magnetic slurry with different dosage of magnetic powder, the saturation magnetisation strength (*B*_s_) of Fe_3_O_4_ is 972.08 times higher than that of the composite cement. The *B*_s_ of the magnetic slurry with 5%, 15%, 25%, and 35% magnetic powder dosage is 25.25, 72.84, 116.99 and 159.37 times of composite cement, respectively. The *B*_s_ of the 35% magnetic powder dosage of the magnetic slurry is 11.53 emu/g, which is 6. 31 times higher than that of the 5% magnetic powder dosage. In terms of magnetisation, the magnetisation of the slurry with 35% magnetic powder doping is 6.89 times higher than that of 5% magnetic powder doping, but only 10.76% of the magnetisation of Fe_3_O_4_. This means that the magnetisation rate of the slurry with more magnetic powder doping is greater. As shown in Fig 8, the saturation magnetisation intensity and magnetisation rate of the magnetic slurry increase linearly as the magnetic powder doping increases.

**Table 1:** Magnetic parameters of slurry with different dosage of magnetic powder.

Materials	Saturation magnetisation (*B*_s_，emu/g)	Coercive magnetic field (*H*_c_，Oe)	Residual magnetisation (*B*_r_，emu/g)	Magnetisation *χ*
Cement	0.07236	103.213	0.00545	2.88 × 10^−5^
Fe_3_O_4_	70.34	86.35	5.869	0.0549
MEC(5%)	1.82691	139.966	0.16376	8.58 × 10^−4^
MEC(15%)	5.27097	98.853	0.46263	0.00251
MEC()25%	8.46594	76.968	0.94669	0.00424
MEC(35%)	11.53227	122.511	1.04409	0.00591

### 3.2. Rheological properties of slurry

The rheological properties of the slurry play a decisive role in its stability and pumpability, and the viscosity is inversely proportional to the spreading distance of the slurry, and the greater the viscosity, the greater the reduction in injectability. Therefore, the study of its rheological properties is of great importance. The performance of the magnetic slurry is affected by the external admixtures and cementitious materials, in addition to the magnetic field effect is also critical. The viscosity is an important parameter for ensuring the stability and anti-segregation performance of the slurry. The yield stress, meanwhile, determines the fluidity of slurry. The rheological properties of magnetic slurry were investigated in relation to magnetic powder doping and magnetic field strength.

#### 3.2.1. Slurry viscosity variation with magnetic field.

**Fig 9:**
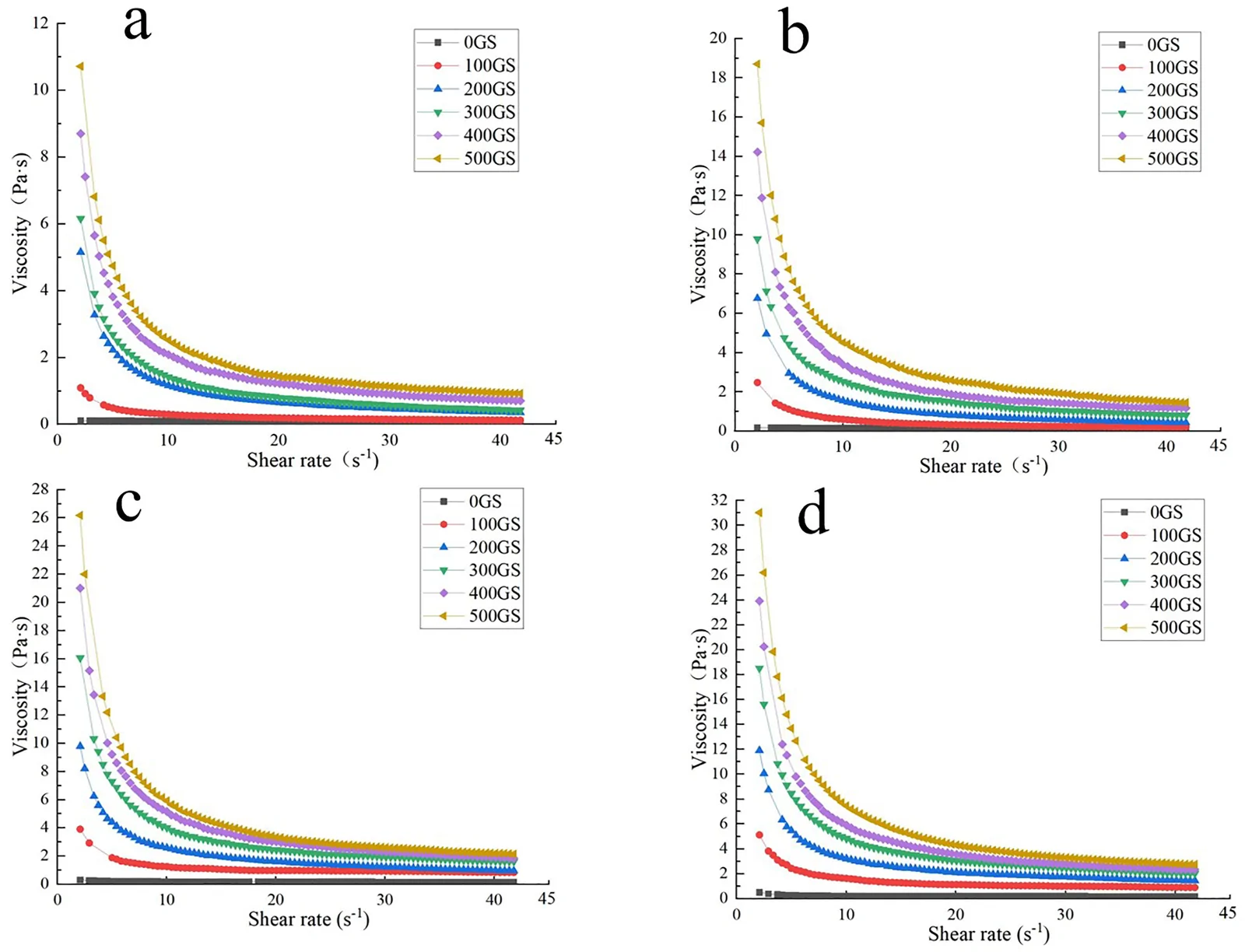
(a) *φ* = 5%. (b) *φ* = 10%. (c) *φ* = 15%. (d) *φ* = 20%. Viscosity change of slurry with different magnetic field strengths.

As demonstrated in Fig 9, in the absence of an applied magnetic field, the viscosity of the slurry exhibits a rapid decrease as the shear rate is increased, eventually reaching a state of gradual stabilisation. The primary reason for this phenomenon is that the flocculation between cement particles is greater than the torque of the rotor before shear. As the shear rate increases, the molecular structure of the flocculation is destroyed, and the free water wrapped in cement particles is released. This process leads to a reduction in the viscosity of the slurry. Following the complete destruction of the flocculating structure, a stabilisation of the slurry’s viscosity is observed.

Before the shear effect is applied, the magnetic particles and polymer chains in the magnetic slurry are arranged in a specific structure due to the applied magnetic field. This arrangement results in a static equilibrium of magnetic chains and a magnetic chain network, which collectively have the lowest energy level within the system. When the shear rotor rotates and produces a vortex in the slurry, the energy of the system gradually increases, causing the original balanced structure to break down. The vortex action instigates the rupture of the magnetic chain and magnetic network in the slurry, thereby forming polar short chains. These polar short chains are easily moved by the vortex action and offer little resistance, resulting in shear thinning phenomenon. After the short chain is sheared during the process of moving with the vortex, the chains collide and reorganize to form a new magnetic chain or magnetic chain network. This configuration is characterised by a state of antagonism and elevated energy levels. When the disruption and reorganisation of the magnetic chains reach an equilibrium state, the viscosity of the slurry no longer varies with increasing shear rate.

**Fig 10:**
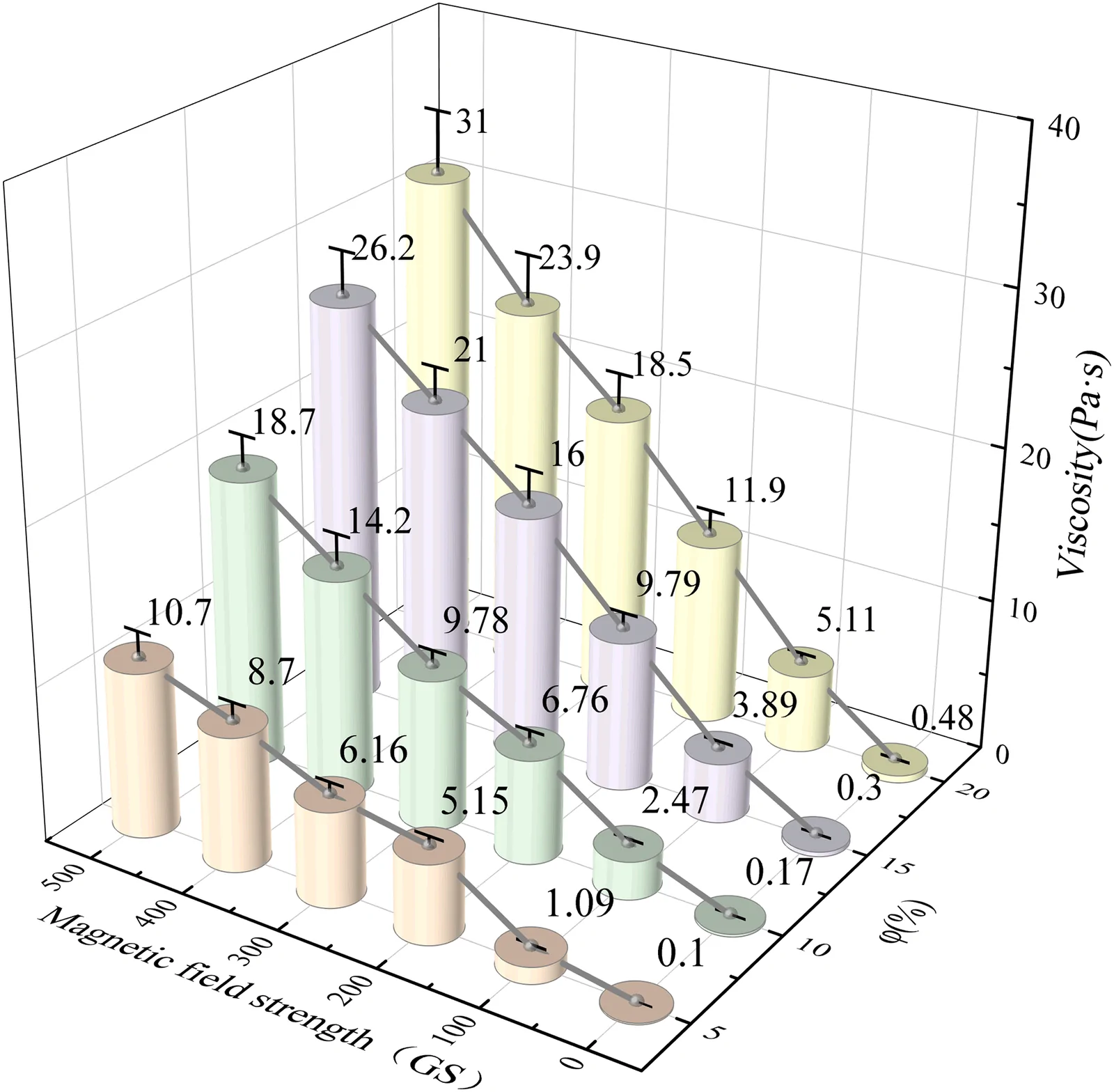
Viscosity variation with magnetic field and magnetic powder dosing.

The measured viscosity of the slurry at a shear rate of 2.09 s^-1^ was taken uniformly for analysis. As shown in Fig 10,when the magnetic field strength is 0 GS, the viscosity of the slurry increases linearly with the dosage of magnetic powder, and the viscosity of 20% magnetic powder dosage is 4.8 times that of 5% dosage. The main reason is that the magnetic particles have a huge specific surface area, which adsorbs and agglomerates with cement particles and hydration products, making the viscosity of the slurry increase. The viscosity of the slurry without magnetic field was 0.1 Pa-s at 5% magnetic powder doping, and with the increase of magnetic field strength (100GS ~ 500GS), the viscosity of the slurry rapidly increased to 1. 09 Pa·s, 5.15 Pa·s, 6.16 Pa·s, 8.7 Pa·s and 10.71 Pa·s. The viscosity of the slurry at 100 GS magnetic field is 10.9 times that of the slurry without magnetic field, and the viscosity of the slurry at 500 GS magnetic field is 107. 1 times that of the slurry without magnetic field, so it is evident that the viscosity of the MEC slurry has a significant time-variation with magnetic field. When the dosage of magnetic powder is 20%, the viscosity of the slurry without magnetic field is 0. 48 Pa·s, and with the increase of magnetic field strength, the viscosity of the slurry gradually increases to 5.11 Pa·s, 11.9 Pa·s, 18.5 Pa·s, 23.89 Pa·s and 31 Pa·s. Furthermore, the viscosity of slurry at a magnetic field strength of 500 GS is found to be 64.8 times greater than in the absence of a magnetic field.

#### 3.2.2. Variation of shear stress with magnetic field strength.

**Fig 11:**
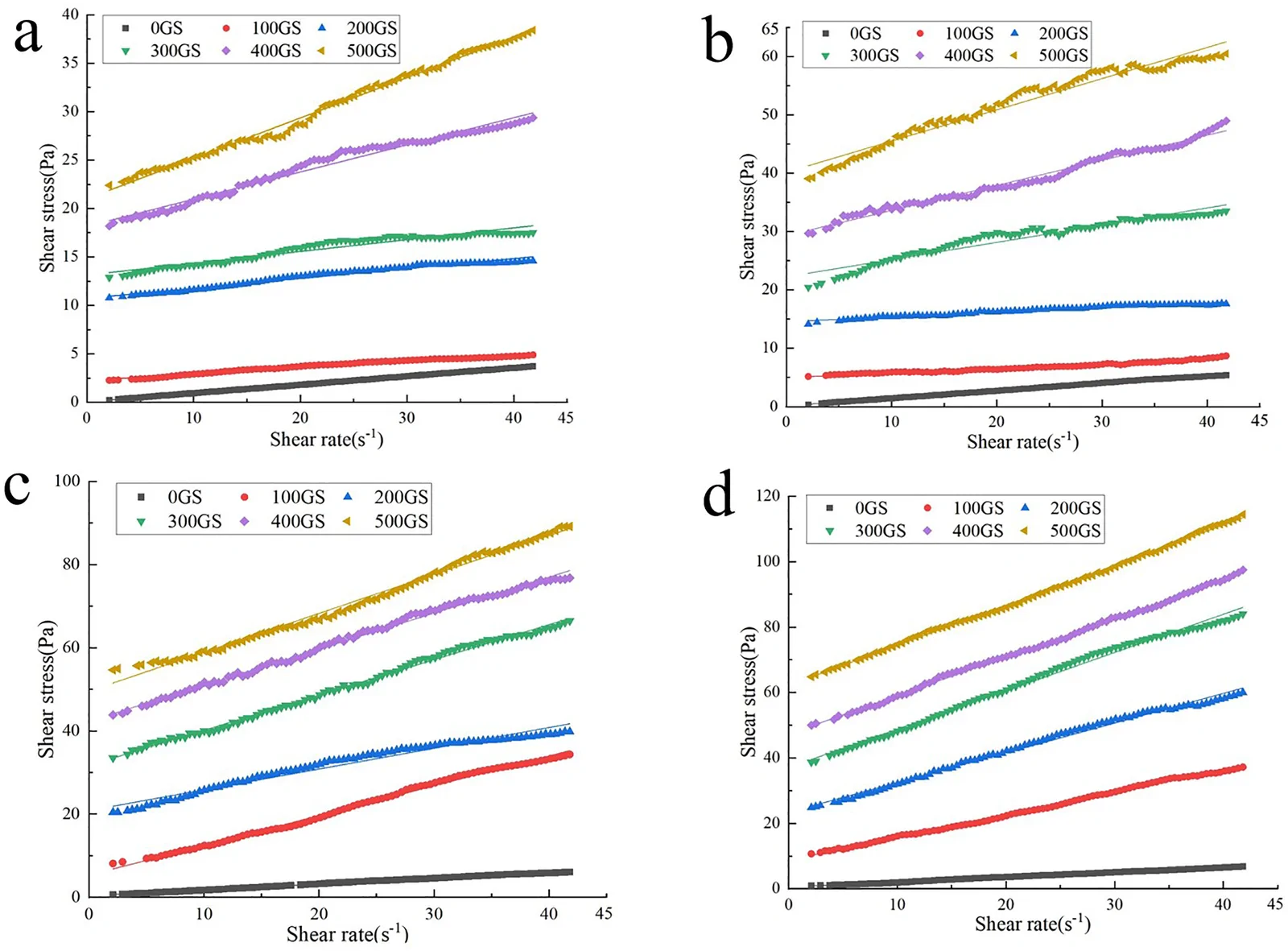
(a) *φ* = 5%. **(b)** ***φ*** **= 10%. (c)** ***φ*** **= 15%. (d)** ***φ*** **= 20%.** Variation of shear stress in slurry with different magnetic field strengths.

The yield stress of regular cement slurry is produced by van der Waals force and inter-particle friction. As shown in Fig 11, the shear stress of magnetic slurry increases gradually with the shear rate. In the absence of a magnetic field, the slurry containing 5% magnetic powder exhibits Newtonian fluid-like behaviour. As the magnetic powder doping increases, the slurry behaves as a Bingham fluid with yield stress, indicating that the increase in magnetic powder doping leads to an increase in the viscosity of the slurry. Under the influence of various magnetic powder dopings, the rheological curves of the slurry were consistent with the Bingham model when the magnetic field was applied. The dynamic yield stress of the slurriy increased with the strength of the magnetic field. When comparing the 10% and 15% doped slurry, it is evident that the magnetorheological response of the 15% doped slurry is more significant at a magnetic field strength of 100 GS. Additionally, the flow curve shows a clear upward slope, indicating an increase in viscosity. The increase in magnetic powder doping expands the source of magnetic particles that form the magnetic force chain, increasing the contact probability of magnetic particles. This enables the continuous replenishment of a new force chain, which in turn provides shear stresses during the process of vortex disruption.

#### 3.2.3. Relation between yield stress and magnetic field strength.

**Fig 12:**
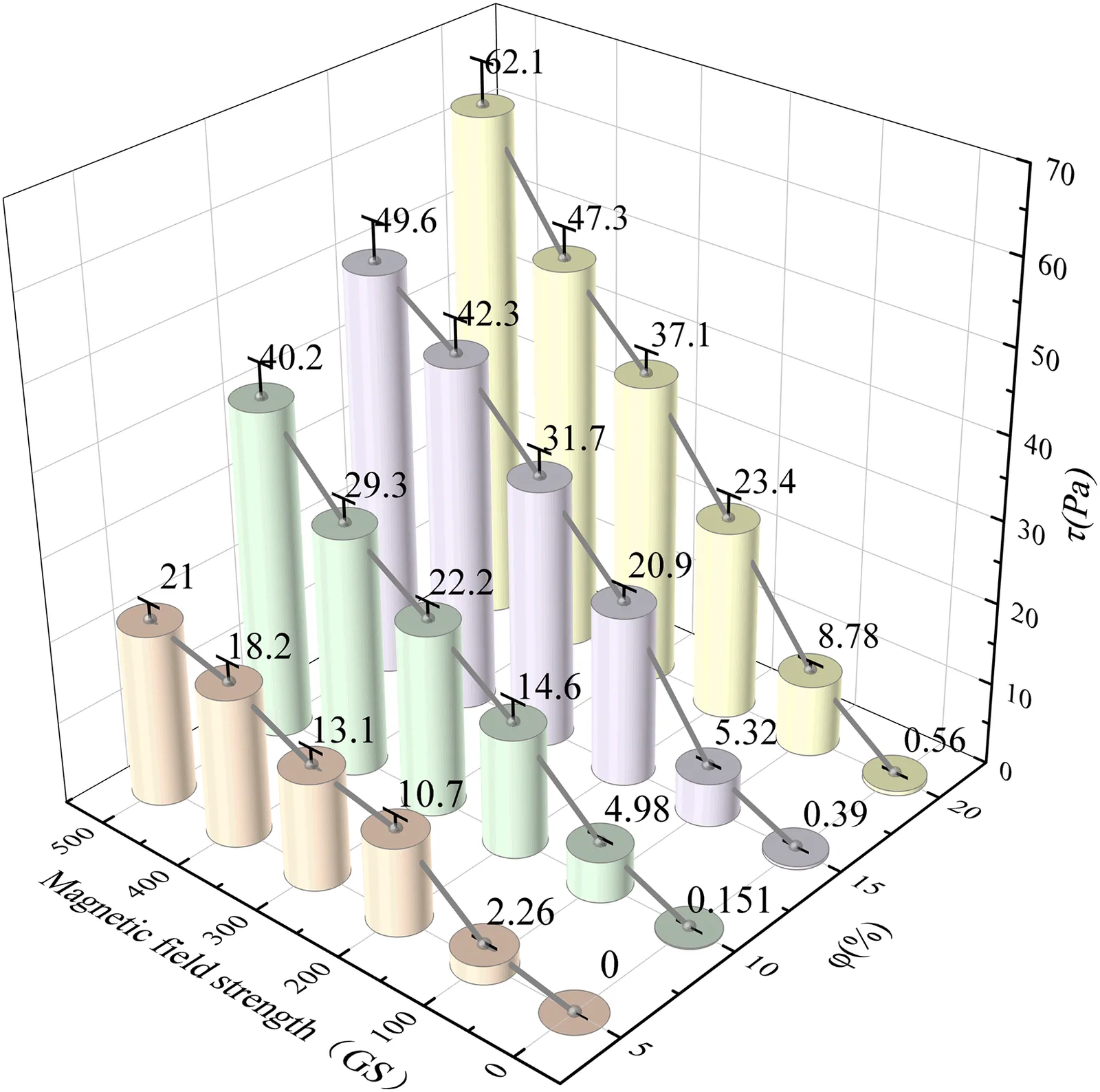
Variation of yield stress with magnetic field strength and magnetic powder dosage.

The yield stresses of magnetic slurry increase with both the magnetic field strength and magnetic powder doping levels. At a magnetic field strength of 0 GS, the dynamic yield stress of 5% doped magnetic powder is 0. The yield force of 20% doped magnetic powder is 3.73 times greater than that of 10% doped and 2.6 times greater than that of 15% doped. When the magnetic field was increased from 0 GS to 500 GS, the yield stress of the 5%, 10%, 15%, and 20% doped magnetic powder increased by 21.01 Pa, 267.8 times,127.13 times, and 110.95 times, respectively. Fig 12 shows that the yield stress growth rate is highest when the magnetic field increases from 100GS to 200 GS. As the magnetic field continues to increase beyond 200 GS, the growth rate of the yield stress decreases for slurry with different magnetic powder dosages. This is mainly due to the fact that when magnetic particles do not form a reticulated or long-chain structure, their shear resistance is lower. However, at a magnetic field strength of 100 GS, the magnetic particles have been observed to form chains or a reticulated arrangement, thereby providing resistance to shear deformation and resulting in a rapid increase in yield stress.

Under varying magnetic field strengths, there exists a linearly increasing relationship between the yield stress of the slurry and the dosage of magnetic powder. The fitting parameters between the viscosity and the magnetic powder dosage under different magnetic field strengths are presented in Table 2. The parameter *k* exhibits a linear relationship with the magnetic field strength *B*, while the intercept *b* is exponentially related to the magnetic field strength *B*. The fitting analysis was conducted separately, and the relationship equations for the parameters *k*, *b*, and the magnetic field strength *B* were derived.

**Table 2:** Slurry yield stress fitting parameters.

*B* (GS)	*k*	*b*	R
0	0.0384	−0.205	0.992
100	0.398	0.36	0.923
200	0.885	6.325	0.976
300	1.625	5.72	0.987
400	2.008	9.175	0.972
500	2.655	10.03	0.979


$$\begin{array}{c} \tau_{\text{0}}=k\varphi +b \end{array}$$ (2)

$$\begin{array}{c} k=0.0053B-0.064 \end{array}$$ (3)

$$\begin{array}{c} b=0.0264B^{0.964} \end{array}$$ (4)

The substitution of Equations (3) and (4) into Equation (2) results in the yield stress τ₀ against the magnetic field strength *B* and the magnetic powder doping *φ*.


$$\begin{array}{c} \tau_{\text{0}}=k\varphi +b=0.0053B⬝\varphi -0.064\varphi +0.0264B^{0.964} \end{array}$$ (5)

### 3.3. Modelling of slurry yield stress variation

**Fig 13:**
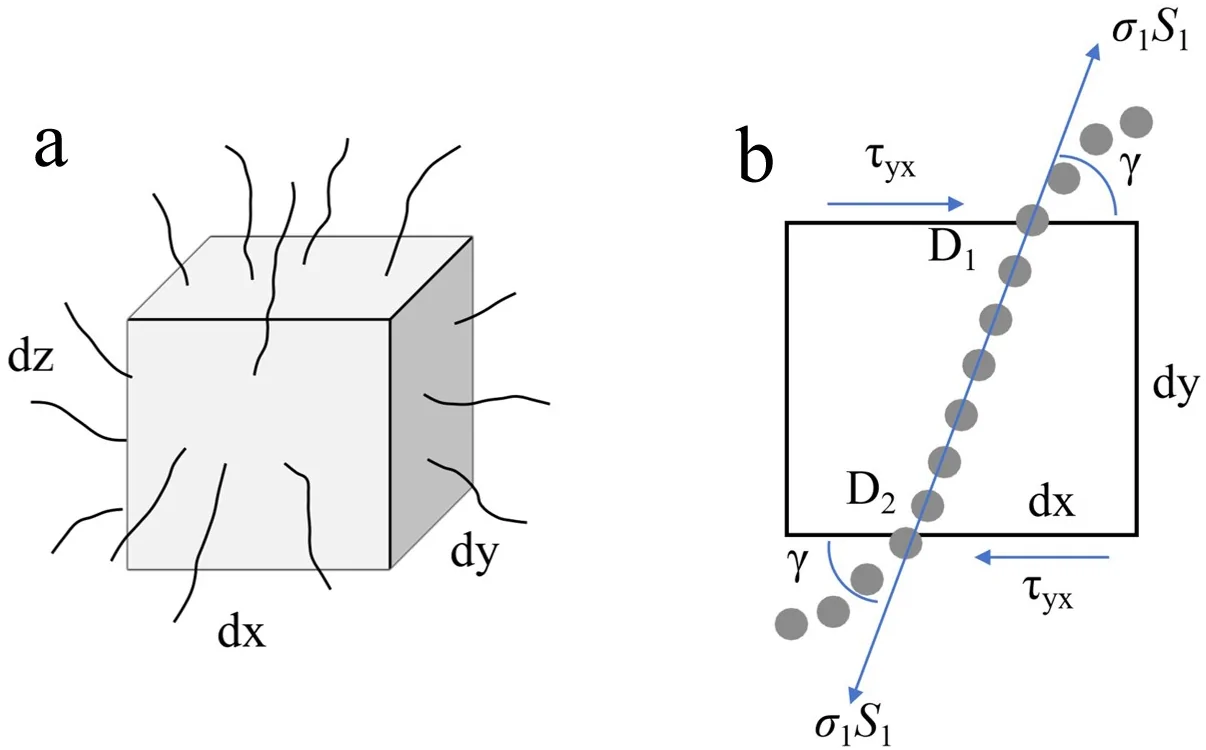
(a) microparticle hexagonal control element. (b) dxdy plane. Schematic representation of a sheared dipole chain.

The magnetic slurry are fluids with microstructured magnetic chains, non-Newtonian fluids, also known as plastic Bingham fluids. The mesh of magnetic particles in the slurry is the main source of yield stress. Fig 13(a) shows a micrometahedron hexahedron with a large number of dipole chains interspersed. Fig 13(b) shows the plane cell analysed, D_1_D_2_ is the magnetic force chain through the dzdx plane, γ is the angle between D_1_D_2_ and τ_yx_, and the shear force is transmitted through the slurry to the force chain D_1_D_2_.

Since the slurry without magnetic powder is itself a Newtonian fluid with negligible yield stress, the stiffness of the slurry is not considered here. According to the law of stress distribution by stiffness, the shear force is all on the magnetic chain. The following equation can be obtained from the equilibrium equation of forces.


$$\begin{array}{c} \tau_{\text{yx}}\text{d}z\text{d}x⬝{\left(cos\gamma \right)}_{\text{e}}={\sum_{i=1}^{dN_{\text{t}}}\left(\sigma_{\text{1}}S_{\text{1}}\right)}_{\text{i}} \end{array}$$ (6)

Where N_t_ is the number of magnetic chains passing through the dzdx plane and (cosγ)e is the mathematical expectation of cosγ.

Magnetic chains are generated by magnetic particles undergoing Brown’s motion. cosγ has a stochastic character and it is assumed that cosγ follow a normal distribution.


$$\begin{array}{c} E\left\{\text{u}\right\}=\int_{x_{\text{1}}}^{x_{\text{2}}}up\left(x\right)\text{d}x=0 \end{array}$$ (7)

The distribution density function p(x) is shown below.


$$\begin{array}{c} p\left(x\right)=\frac{\text{1}}{\sqrt{2\pi }\sigma }e^{-\frac{\text{1}}{2\sigma^{\text{2}}}{\left[cos\gamma -{\left(cos\gamma \right)}_{\text{e}}\right]}^{\text{2}}} \end{array}$$ (8)

If the attraction within a dipole pair is used instead of the attraction between two neighbouring dipoles in a magnetic chain, the following equation is obtained.


$$\begin{array}{c} {\left(\sigma_{\text{1}}S_{\text{1}}\right)}_{\text{i}}\leq (F_{d,r}{)}_{\text{i}}=-\frac{3\mu_{\text{0}}}{4\pi r^{\text{4}}}m_{\text{d},i}m_{\text{d},i+1}\left[3cos\left(\theta_{\text{1}}-\varphi \right)cos\theta_{\text{1}}-cos\varphi \right]{\vec{r}}^{\text{0}} \end{array}$$ (9)

The yield stress of the magnetic slurry is finally obtained.


$$\begin{array}{c} \tau_{\text{s}}=n_{\text{t}}\frac{3\mu_{\text{0}}}{\pi r^{\text{4}}}m_{\text{d}}^{\text{2}}=n_{\text{t}}\frac{\mu_{\text{0}}}{4r_{\text{0}}}\chi B \end{array}$$ (10)

From Eq. (10) it can be seen that the yield stress of the slurry is related to the number of magnetic chains per unit area *n*_t_, the external magnetic field strength *B*, the radius of the magnetic particles *r*_0_ and the magnetisation rate *χ*, which are proportional to the number of magnetic chains *n*_t_, the magnetisation rate *χ* and the external magnetic field strength *B*, and inversely proportional to the radius of the particles r_0_.

### 3.4. Magnetic particle distribution pattern

#### 3.4.1. Schematic distribution of magnetic particles in the slurry.

**Fig 14:**
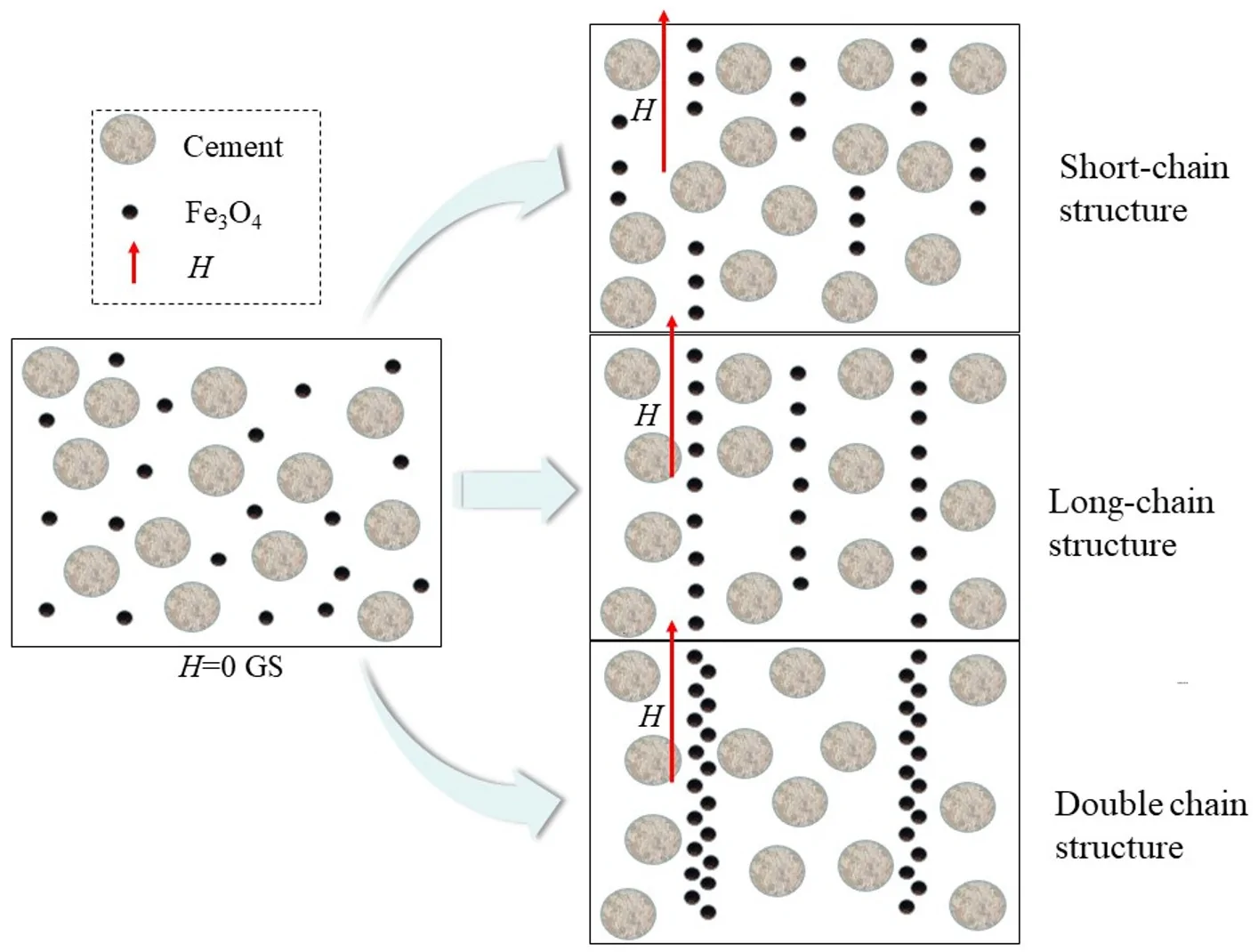
Schematic diagram of the chain formation pattern of magnetic particles in the slurry.

The primary cause of the viscosity transient of the MEC slurry is the magnetically induced chain formation of the magnetic particles, which can undergo a rapid transformation, within milliseconds, from a randomly disordered state into an ordered structure such as a chain distributed along the direction of the external magnetic field. This transformation is both controllable and reversible between a fluid and a solid-like substance. The magnetic particles rotate and move in the same direction as the external magnetic field due to the magnetic force and moment. The Fig 14 demonstrates the magnetisation process of Fe_3_O_4_ magnetic particles forming chains in a cement-based magnetic slurry. In the absence of a magnetic field, the distribution of magnetic particles within the slurry is uniform. As the strength of the magnetic field increases, the particles form short chains, long chains, double chains, and bundled chains.

#### 3.4.2. Distribution morphology of magnetic particles with magnetic field.

**Fig 15:**
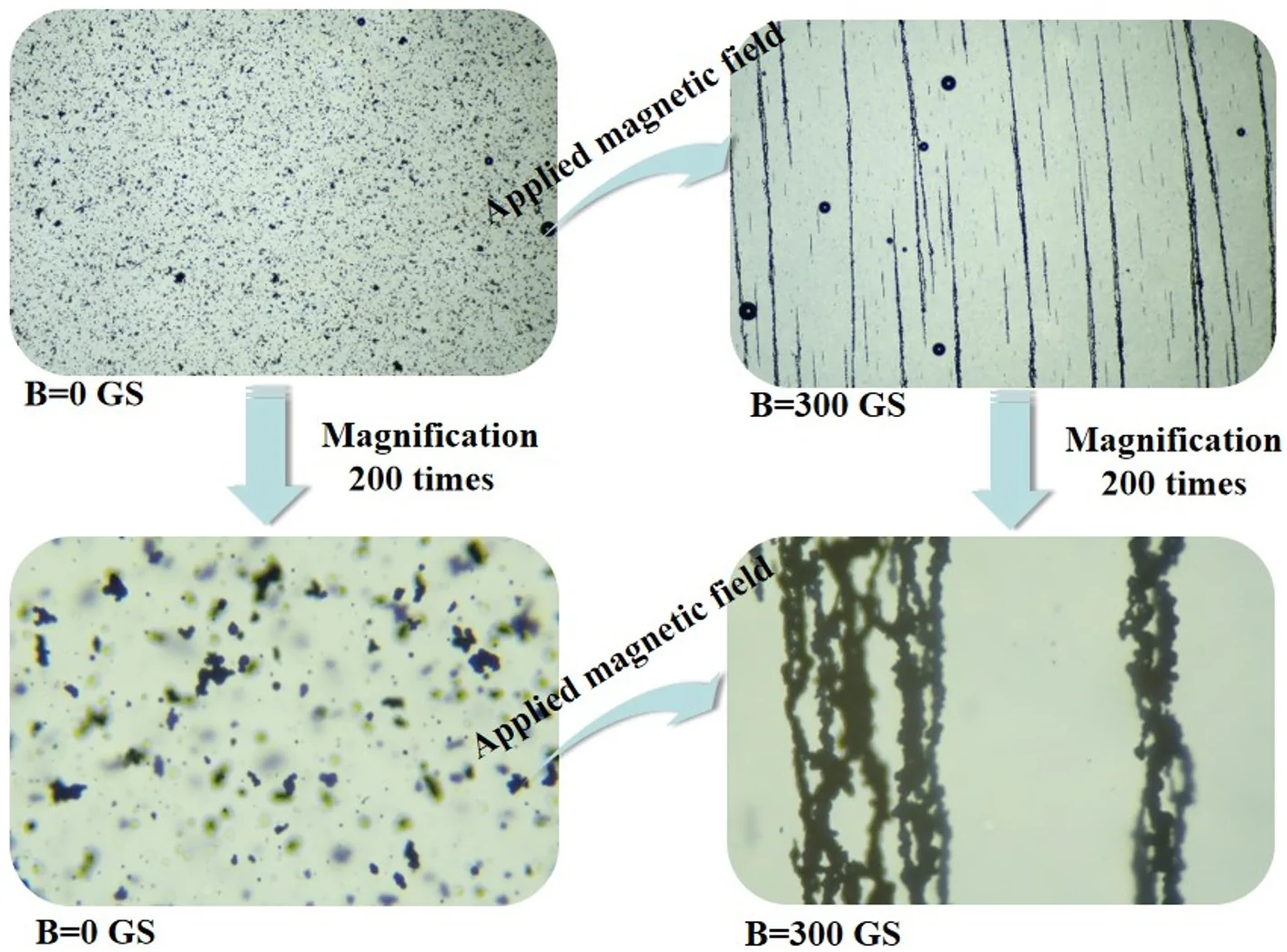
Patterns of the distribution of magnetic chains in the presence of a magnetic field.

As demonstrated in Fig 15, in the absence of a magnetising field, the magnetic particles are distributed uniformly throughout the slurry. The paste contains both individual particle monomers and aggregates formed by multiple particle monomers. When a 300 GS magnetic field is applied, the magnetic moments of the particle monomers in the aggregates are randomly oriented, resulting in some monomers being weakly or neutrally magnetic. Magnetic chains are formed from both monomers and aggregates of magnetic particles. The magnetic chains doped with 3% are closely spaced with contact laps between them. Upon magnification of 600 times, it is evident that each bundle chain is a single chain with multiple magnetic particles overlapping, and the average magnetic chain spacing is 16.5 μm. The probability of inter-particle attraction increases, and the number of magnetic particle chains and their proximity to each other increases. This leads to mutual attraction movement and aggregation, resulting in the formation of a magnetic mesh structure.

#### 3.4.3. Patterns of three-dimensional spatial distribution of magnetic chains.

In the experimental setup, the magnetic field strength was set to 5000 GS, with the magnetic field directed perpendicular to the bottom of the test specimen. The specimens used for the CT testing were cured for a strictly controlled period of 7 days. To ensure the accuracy of the test results, the specimen surfaces were finely polished to achieve a smooth finish. The specimens were cubic in shape, with a volume of 343 mm³.

**Fig 16:**
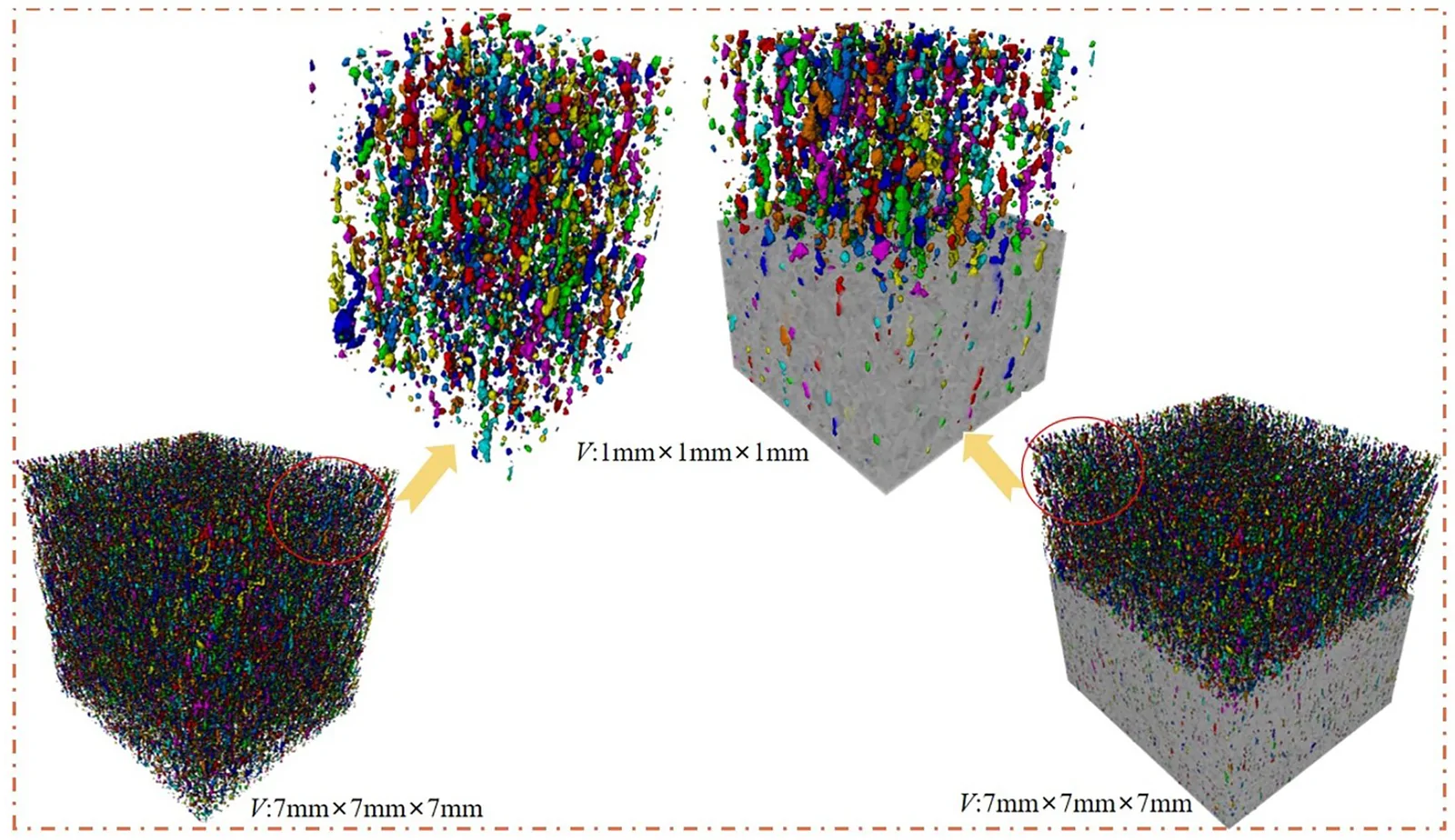
Three-dimensional distribution pattern of magnetic chains of micron-sized magnetic particles.

**Fig 17:**
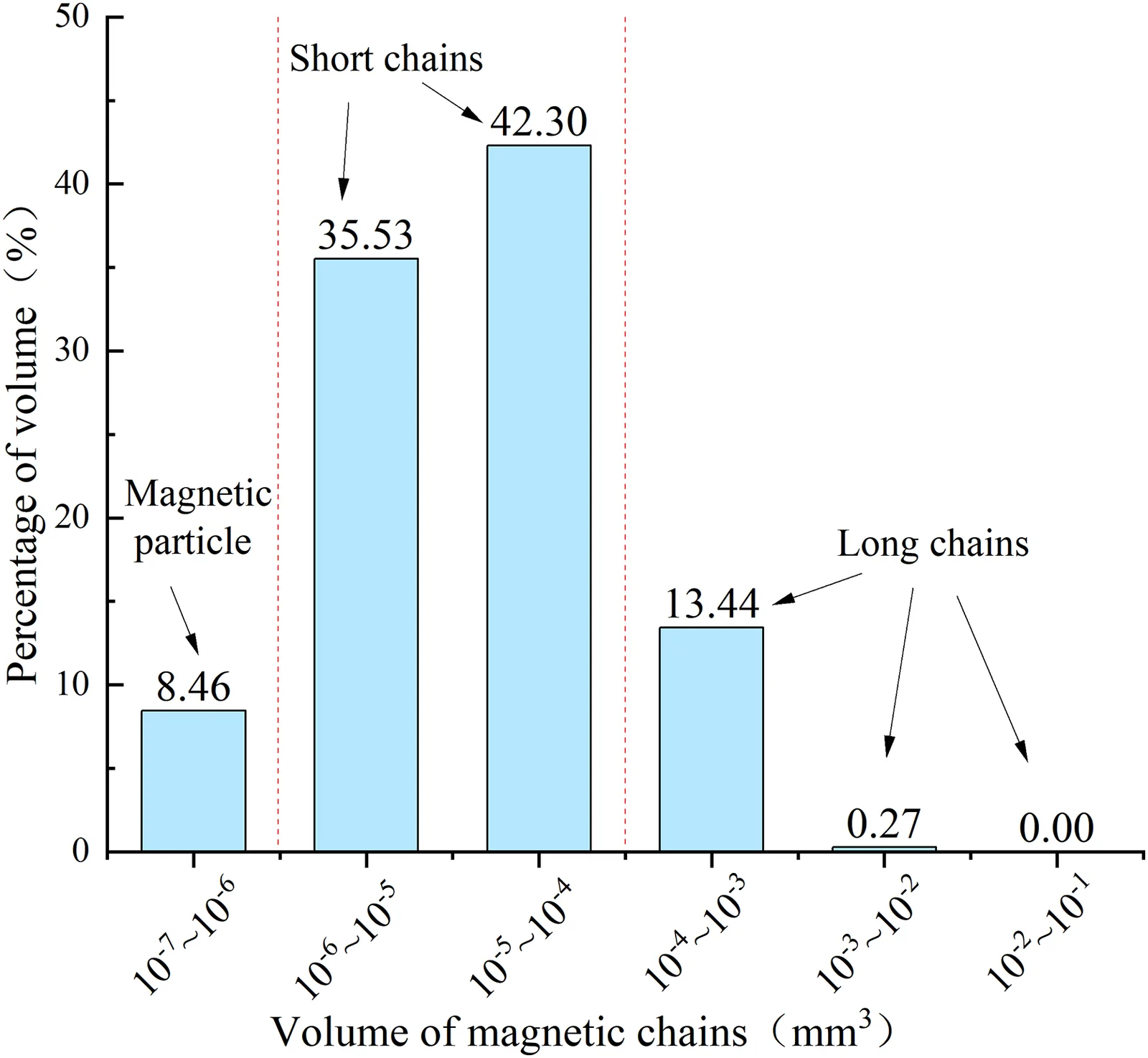
Volume distribution of magnetic chains.

A high-precision CT scanning method was used for 3D reconstruction after threshold segmentation of the image, as shown in Fig 16, which shows the 3D distribution morphology of micron-sized magnetic particles under the action of a magnetic field, and the magnetic powder doping of the slurry is 25%. There are 137858 magnetic chains in the small cubic magnetic plasma sample with a side length of 7 mm, and the volume distribution of the magnetic chains ranges from 6.336 × 10^−7^ mm^3^ to 0.023556 mm^3^, with a total volume size of 7.365 mm^3^, and for the volume of the size of the order of 10^−7^ mm^3^ is the volume of individual magnetic particles, which accounts for the ratio of the total volume of the chain of magnetic particles to the total volume of the magnetic chains is 8.46%. As shown in Fig 17, the volume fraction of shorter magnetic chains is 77.83% and the fraction of longer chains is 13.71%. Most of the shorter chains are aligned along the direction of the magnetic field and there are fewer chain links in the transverse direction in three-dimensional space.

#### 3.4.4. Distribution of magnetic particle chains with a particle size of millimetres.

**Fig 18:**
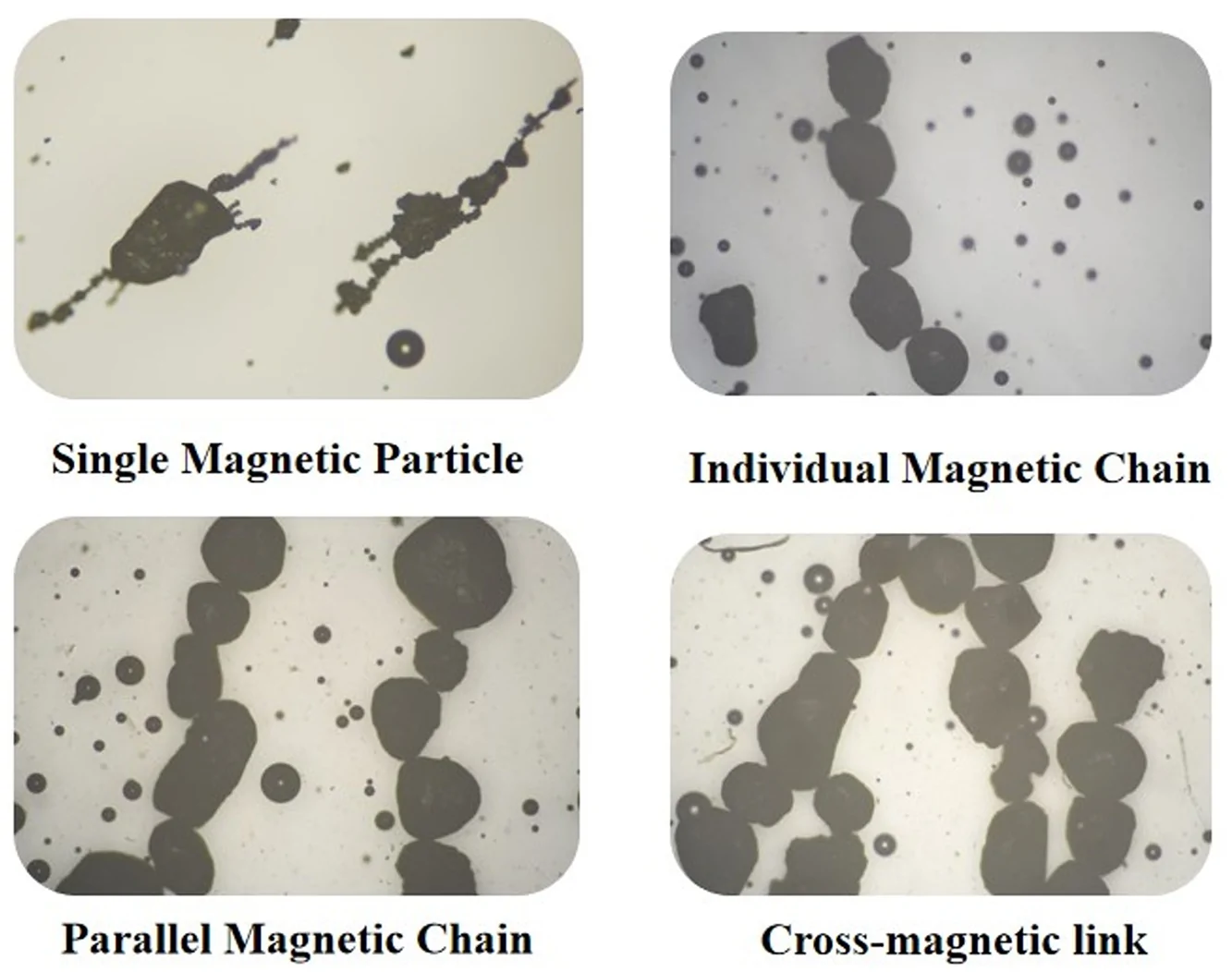
Magnetotelluric chain diagram of millimetre-sized magnetic particles.

**Fig 19:**
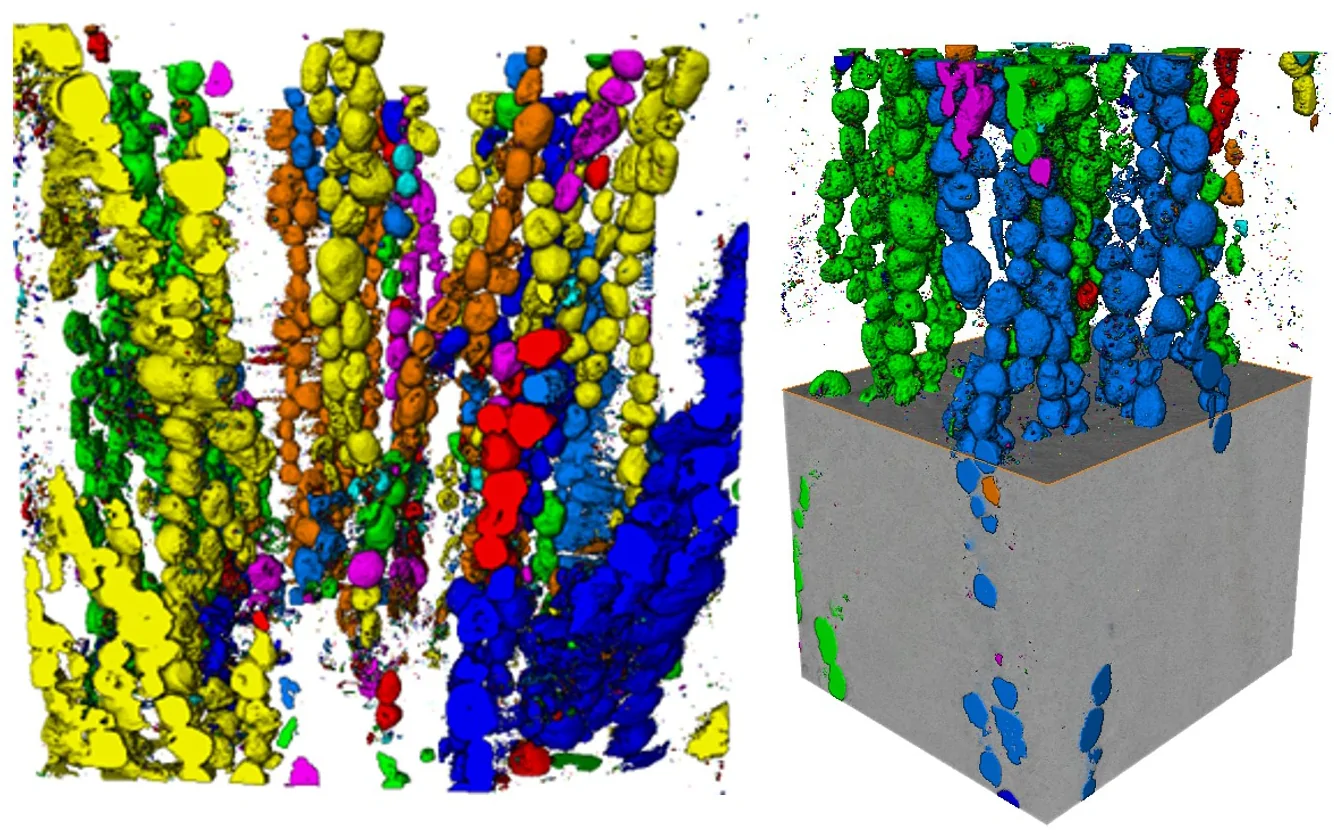
Three-dimensional distribution pattern of magnetic chains of millimetre-sized magnetic particles.

In order to observe the particle attraction motion pattern more intuitively under the microscope, the millimetre-sized magnetic particles were placed in a 300 mPa·s transparent resin slurry. As shown in Fig 18, a single magnetic particle attracts and aggregates tiny chains of magnetic particles, and the large particles overlap each other along the direction of the long axis of the particles to form a chain-like structure. Some of the magnetic chains are parallel to each other and some of them cross over each other to form a reticulated chain. As can be seen in Fig 19, the magnetic powders, which are millimetre-sized, are distributed in clusters with tight interparticle bonds. In the magnetic field region, the magnetic chains wrap around the entire sample.

### 3.5. Magnetic slurry scour resistance

Previous research employed a comprehensive weighted scoring method and an optimisation decision model to determine the optimal mix ratio for MEC slurry under the combined influence of five factors: workability, compressive strength, tensile strength, loss rate of cementitious materials, and adsorption efficiency. The results indicated a poly-to-ash ratio of 0.03, a water-to-ash ratio of 0.4, a magnetic powder content of 20%, a flocculant content of 3%, and a water-reducing agent content of 0.2%. As shown in Table 3, in this scour resistance test, tests were conducted using this mix design as a reference, with adjustments made to the magnetic powder content.

**Table 3:** Proportions of the slurry.

Name	Composite cement		Water (g)	Epoxy resin(g)	Magnetic powder (g)	Flocculant(g)	Water-reducing (g)
	PO(g)	SAC(g)					
No. 1	420	180	240	18	0	18	1. 2
No. 2	420	180	240	18	30	18	1. 2
No. 3	420	180	240	18	60	18	1. 2
No. 4	420	180	240	18	90	18	1. 2
No. 5	420	180	240	18	120	18	1. 2

**Fig 20:**
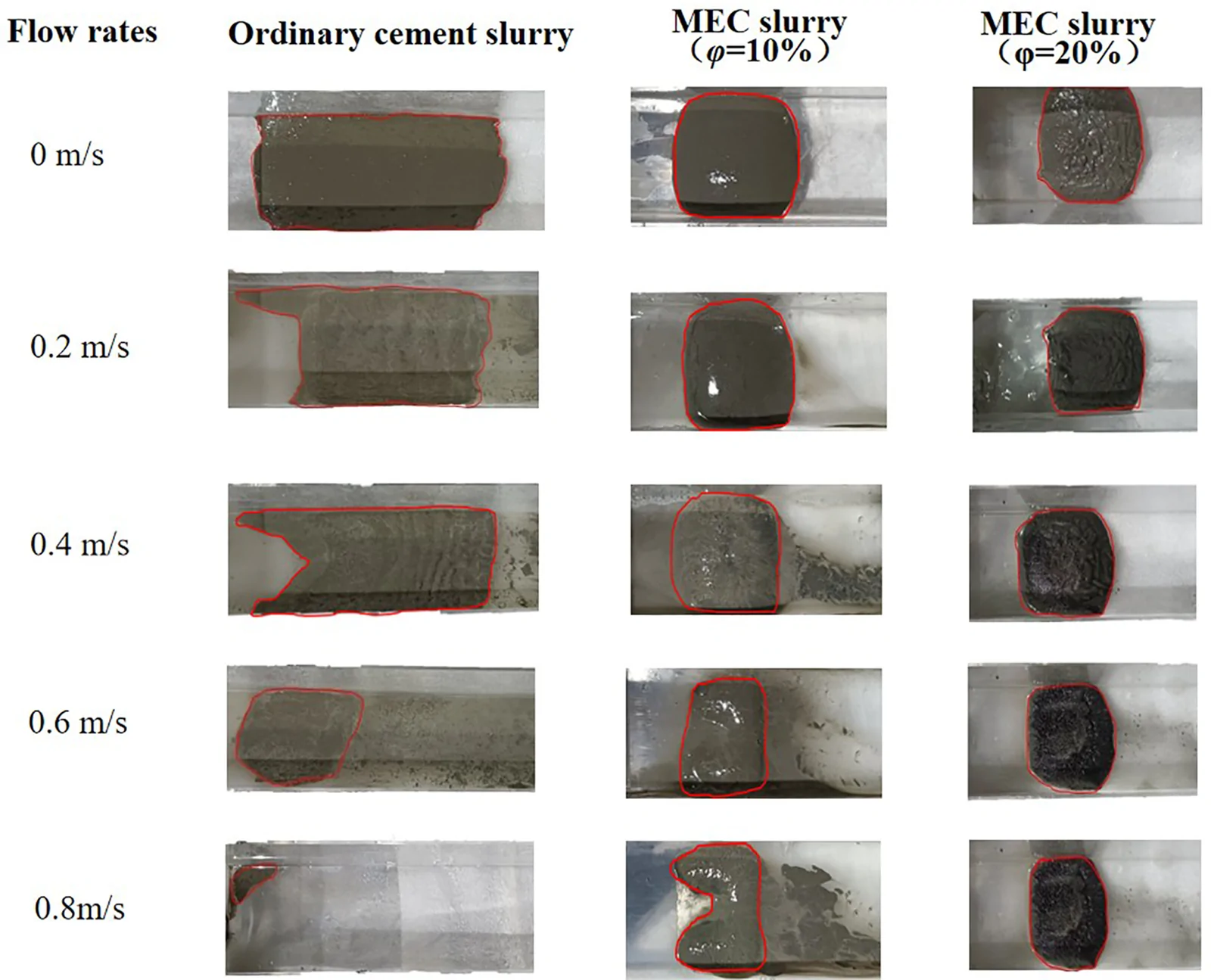
Morphology of the slurry after washing.

As shown in Fig 20, a comparison of the morphology of the slurry following flushing with ordinary epoxy resin cement slurry and magnetic epoxy resin slurry reveals notable differences. The gravity-fed ordinary slurry flows and expands in all directions, while the magnetic slurry exhibits convergence under the influence of magnetic induction. Under the scouring effect of 0.2 m/s dynamic water flow velocity, part of the ordinary slurry is lost with the water flow, and part of the slurry thickness is reduced under the effect of the water flow and gradually expands and flows in the direction of the water flow. In the flow rate of 0.6m/s, the slurry retention rate of 44.2%, as the water flow continues to increase, the slurry is almost no retention. The morphology of the magnetic slurry did not change significantly with an increase in water flow. The magnetic slurry retention rate decreased mainly because the high-velocity water washed away the cement gel adhering to the magnetic powder on the slurry’s surface.

**Fig 21:**
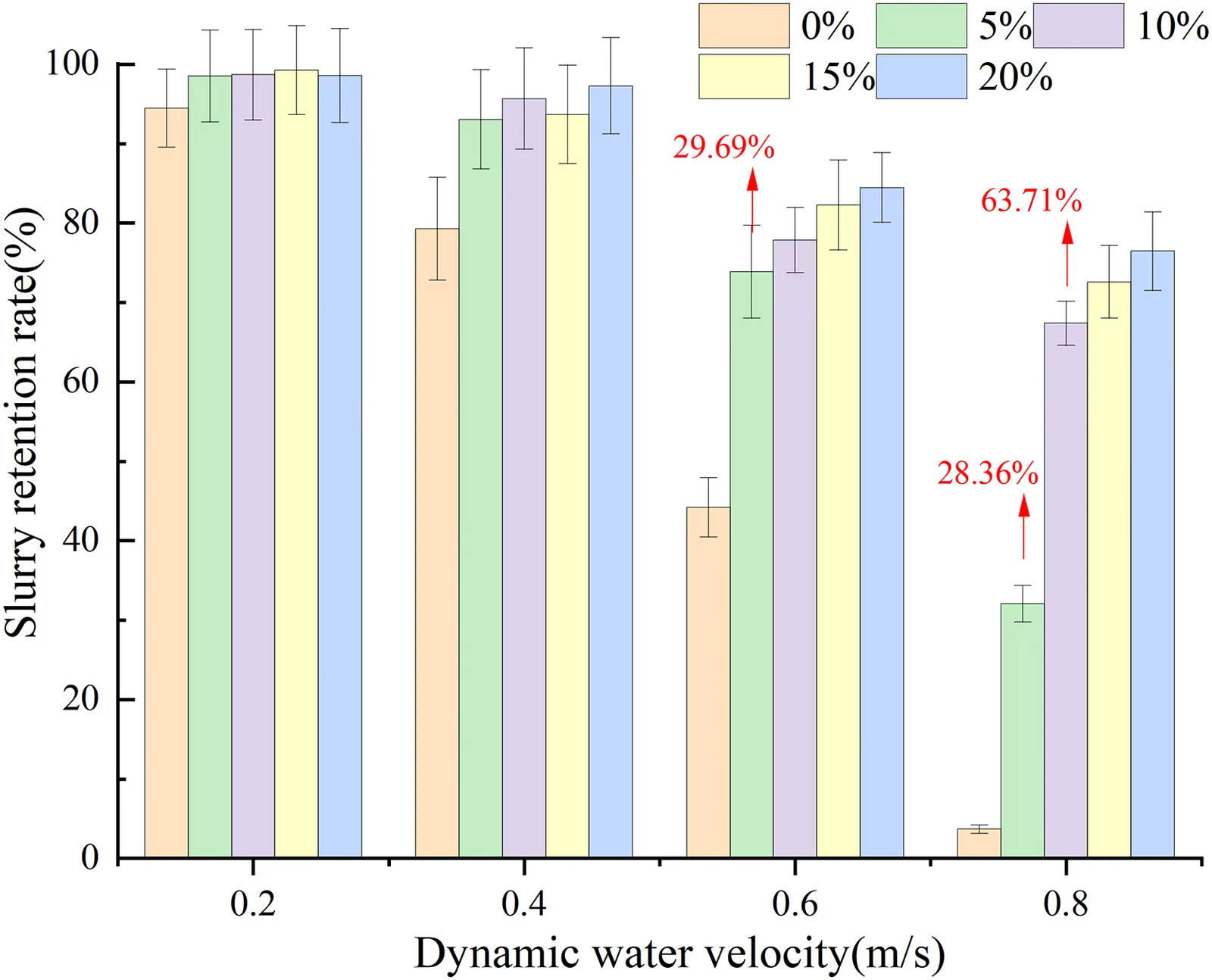
Slurry retention at different magnetic powder dosage and dynamic water flow rates.

**Fig 22:**
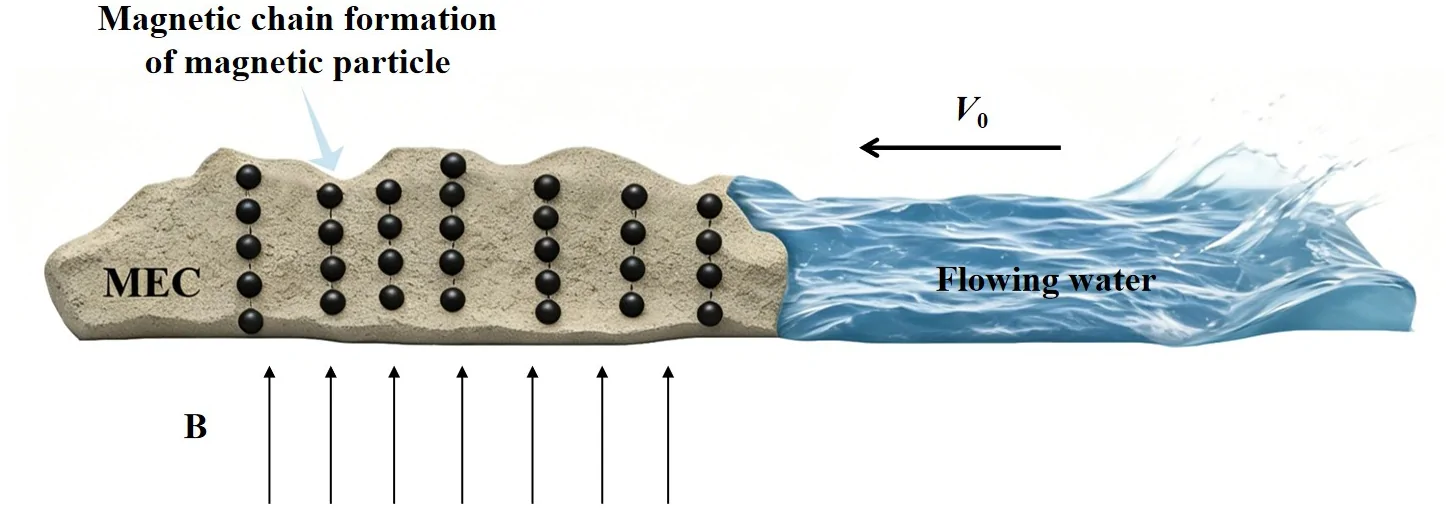
Schematic diagram of slurry anti-erosion.

As shown in Fig 21, the slurry retention rate gradually decreased with the increase of flowing water flow rate. For ordinary epoxy resin cement slurry doped with 0% magnetic powder, the retention rate of slurry decreased rapidly with the increase of flowing water flow rate, and the retention rate of the slurry was only 3.7% at a flow rate of 0.8 m/s. When 5% of magnetic powder was added, the retention rate of slurry was greatly improved under the action of 2000GS magnetic field, and the retention rate of slurry was increased by 28.36% under the condition of 0. 8m/s moving water flow rate. When the magnetic powder dosage was 20%, the retention rate of slurry was 76.5% under 0.8 m/s flow rate, which was 20.67 times higher than that of ordinary epoxy resin cement slurry. Furthermore, as the magnetic powder dosage was increased in a gradual manner, the discrepancy in the slurry’s retention rate diminished progressively. In the dynamic water environment, the ordinary epoxy resin cement slurry has low viscosity of the slurry at the early stage of hydration, and it cannot rapidly form a gel structure under the dynamic water scouring effect, which leads to a low retention rate of slurry. As shown in Fig 22, the viscosity of magnetic slurry under the action of magnetic field increases instantly to tens to hundreds of times, and the shear strength of the slurry under the action of magnetic particles forming chains increases dramatically, which greatly improves the retention rate of the slurry.

### 3.6. Micro-morphological analysis of the slurry

#### 3.6.1. SEM hydration product analysis.

**Fig 23:**
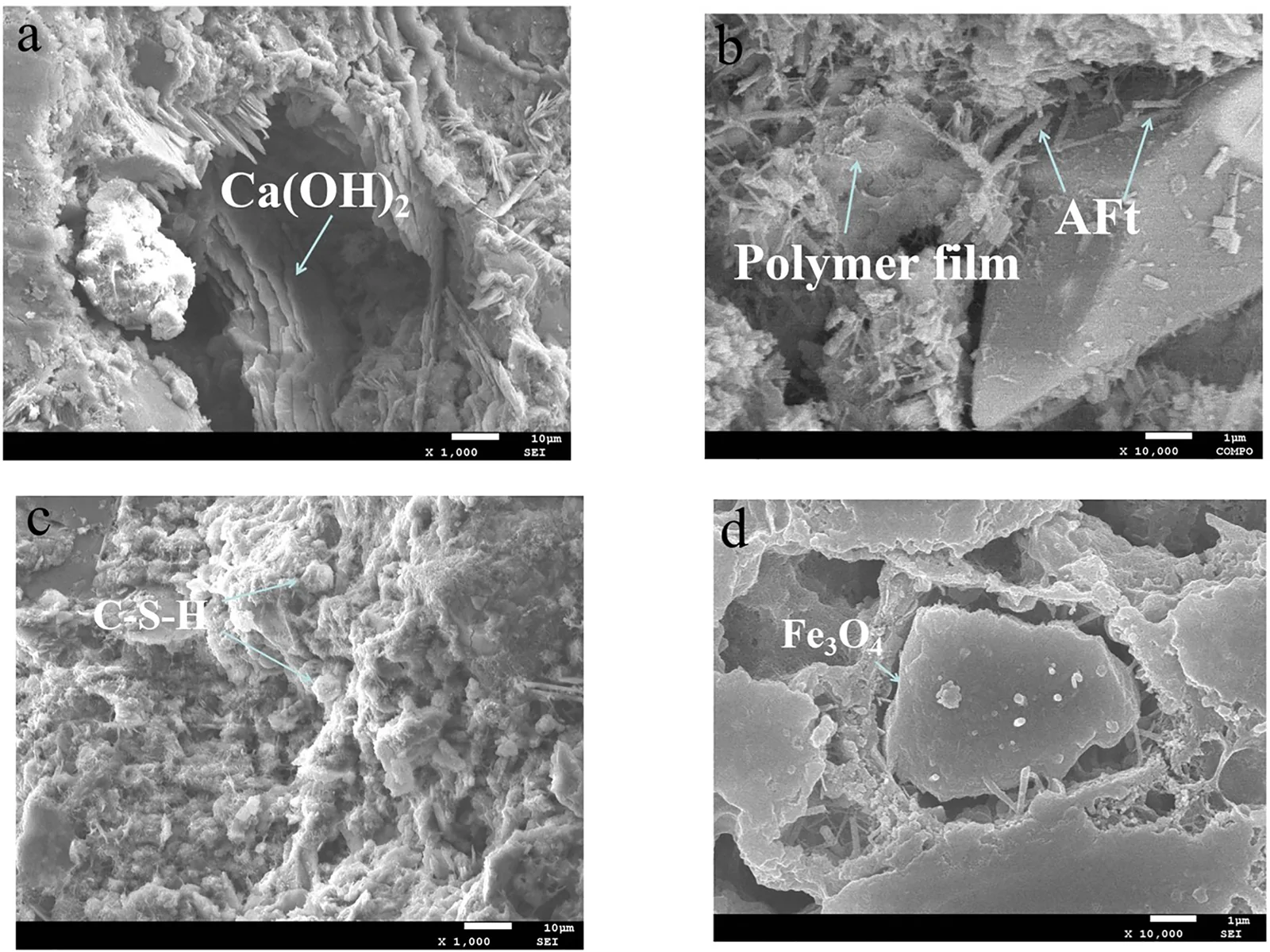
(a) Ca(OH)_2_. (b) Polymer film and AFt. (c) C-S-H. (d) Fe_3_O_4_. Microscopic morphology of different hydration products of MEC slurry.

As demonstrated in Fig 23, the hydration product Ca(OH)_2_ is distributed in the form of flakes. The epoxy resin filling of the pores between the AFt crystals and bonding of the interlayers improves the stability of the crystal structure. The surface of C-S-H gel is coated with a thin layer of epoxy curing compound to form an organic-inorganic composite gel interface with inorganic hydrogel and organic polymer film. As the core strength phase of cement paste, the typical micro morphology of calcium silicate hydrate (C-S-H) is a three-dimensional gel network structure with flocculent and clustered interwoven. The Fe_3_O_4_ was wrapped in epoxy resin and cement hydration products.Under the action of external magnetic field, Fe_3_O_4_ particles migrate and gather directionally driven by magnetic force, which can control the slurry compactness and pore structure.

## 4. Conclusion

This study investigates the influence of Fe_3_O_4_ on the rheological properties of cement slurry, reveals the time-dependent evolution of the slurry’s viscosity under an applied magnetic field, and elucidates the mechanism of viscosity enhancement based on the microstructural evolution of magnetic particle chains, thereby providing a theoretical foundation for the engineering applications of cement-based magnetic slurries.

(1) Magnetisation rate and saturation magnetisation intensity are positively related to magnetic powder dosing. The saturation magnetisation intensity of Fe_3_O_4_ is 972.08 times that of the composite cement and 6.1 times that of the 35% magnetic powder dosing magnetic slurry. The magnetisation rate of the 35% magnetic powder dosing slurry is 10.76% that of Fe_3_O_4_ and 6.89 times that of the 5% magnetic powder dosing, which indicates that the greater the magnetic powder dosing, the greater is the magnetisation rate of the slurry.
(2) The magnetic slurry viscosity has a significant time-variation with the magnetic field, the rheological curves of the slurry under the effect of different magnetic powder doping are in accordance with the Bingham model, and the dynamic yield stress of the slurry increases with the increase of the magnetic field strength. The viscosity of the slurry with a magnetic field of 500GS is 107.1 times higher than that with no magnetic field.
(3) The magnetogenic chain formation mechanism of the slurry was revealed. The viscosity transient mechanism is that the magnetic particles and polymer chains within the magnetic slurry are arranged in a certain structure, and the magnetic chains and the magnetic chain network are in static equilibrium to provide shear strength.
(4) With a flow rate of 0. 8 m/s and 20% magnetic powder doping, the slurry retention rate was as high as 76.5%. This was 20.67 times higher than that of an ordinary epoxy resin cement slurry.
(5) The hydration products were wrapped by the epoxy curing products, which served to fill the pores of slurry, improving the compactness of the paste. Under the action of magnetic field, the Fe_3_O_4_ was wrapped in epoxy resin and cement hydration products.
